# Chalcone‐Supported Cardiac Mesoderm Induction in Human Pluripotent Stem Cells for Heart Muscle Engineering

**DOI:** 10.1002/cmdc.202100222

**Published:** 2021-08-31

**Authors:** Farah S. Raad, Taukeer A. Khan, Tilman U. Esser, James E. Hudson, Bhakti Irene Seth, Buntaro Fujita, Ravi Gandamala, Lutz F. Tietze, Wolfram-Hubertus Zimmermann

**Affiliations:** ^1^ Institute of Pharmacology and Toxicology University Medical Center Georg-August-University Göttingen Germany; ^2^ DZHK (German Center for Cardiovascular Research) – Partner site Göttingen Göttingen Germany; ^3^ Institute of Organic and Biomolecular Chemistry Georg-August-University Göttingen Germany; ^4^ Cluster of Excellence “Multiscale Bioimaging: from Molecular Machines to Networks of Excitable Cells” (MBExC) Georg-August-University Göttingen Germany; ^5^ DZNE (German Center for Neurodegenerative Diseases) – Partner site Göttingen Göttingen Germany; ^6^ Fraunhofer Institute for Translational Medicine and Pharmacology (ITMP) Göttingen Germany

**Keywords:** human pluripotent stem cells, cardiac differentiation, BMP signaling, chalcones, small-molecule screening, tissue engineering

## Abstract

Human pluripotent stem cells (hPSCs) hold great promise for applications in cell therapy and drug screening in the cardiovascular field. Bone morphogenetic protein 4 (BMP4) is key for early cardiac mesoderm induction in hPSC and subsequent cardiomyocyte derivation. Small‐molecular BMP4 mimetics may help to standardize cardiomyocyte derivation from hPSCs. Based on observations that chalcones can stimulate BMP4 signaling pathways, we hypothesized their utility in cardiac mesoderm induction. To test this, we set up a two‐tiered screening strategy, (1) for directed differentiation of hPSCs with commercially available chalcones (4’‐hydroxychalcone [4’HC] and Isoliquiritigen) and 24 newly synthesized chalcone derivatives, and (2) a functional screen to assess the propensity of the obtained cardiomyocytes to self‐organize into contractile engineered human myocardium (EHM). We identified 4’HC, 4‐fluoro‐4’‐methoxychalcone, and 4‐fluoro‐4’‐hydroxychalcone as similarly effective in cardiac mesoderm induction, but only 4’HC as an effective replacement for BMP4 in the derivation of contractile EHM‐forming cardiomyocytes.

## Graphical abstract

Have a little heart: A screen for mesoderm inducing chalcones in human pluripotent stem cell cultures identified 4’‐hydroxychalcone (4’HC) as an effective replacement for bone‐morphogenetic protein 4 (BMP4) in supporting the derivation of engineered heart muscle (EHM)‐formation competent cardiomyocytes.

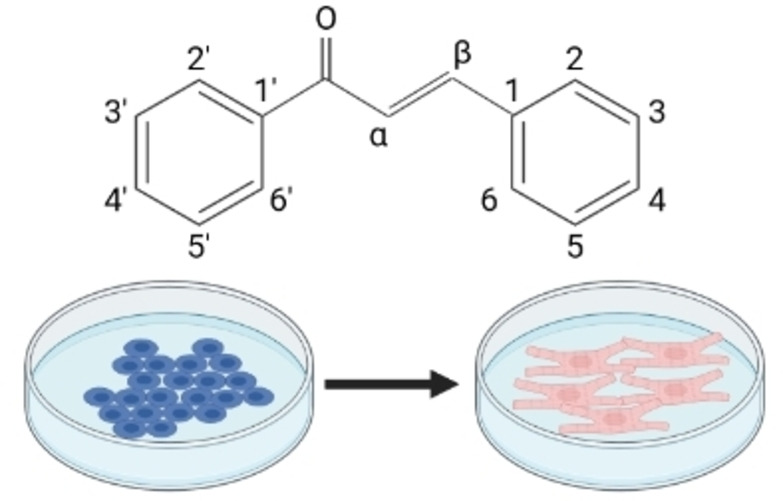

## Introduction

Bone morphogenetic proteins (BMPs), in particular BMP4, have been identified as important factors in the directed differentiation of human pluripotent stem cells (hPSCs) towards the lateral plate (cardiac) mesoderm.^[1]^ As a member of the transforming growth factor‐β (TGF‐β) superfamily, BMPs activate regulatory SMAD transcription factors (R‐SMADs), specifically SMAD 1,5 and 8.^[2]^ Upon their phosphorylation, R‐SMADs oligomerize with a common Co‐SMAD (SMAD 4) and translocate to the nucleus, to regulate the expression of BMP target genes.[^2b^, ^3^] The role of BMP‐SMAD signaling is well documented for embryogenesis, and in particular for cardiac mesoderm formation.^[4]^ In the developing embryo, BMPs are secreted from the extraembryonic mesoderm, creating morphogenic BMP gradients, which in a concentration, spatial, and time dependent manner guide progenitor cell differentiation towards the cardiac mesoderm.^[5]^ Based on observations from embryonic heart development, directed differentiation protocols employing BMP‐receptor activation have been developed in mouse and human PSC models.[^4c^, ^6^] In line with these observations, we have recently found that a combination of Activin‐A, BMP4, CHIR99021, and FGF2 (ABCF‐protocol) supported cardiac mesoderm formation in all tested hPSC lines (including embryonic and induced pluripotent stem cells), with subsequent applicability of the derived cardiomyocytes in the engineering of human heart muscle.^[7]^

The use of BMP4 in cardiac differentiation protocols is hampered by batch‐to‐batch variability, limited stability, and high costs. Hence, small molecular replacements for BMP4 are highly sought after to stabilize the differentiation process at reduced costs. Chalcones have been reported to activate BMP signaling pathways.^[8]^ Here we lay out a two‐tiered screening strategy for the identification of small molecule replacements for BMP4 in directed cardiac mesoderm induction in hPSC for subsequent cardiomyocyte derivation. As a prototypical hPSC, we employed the well characterized human embryonic stem cell line (HES2).[^7^, ^9^] The screening strategy included quantitative (cardiomyocyte yield) and most importantly functional tissue level (cardiomyocyte self‐organization into contractile EHM) read‐outs. The latter are particularly important, to ensure tissue‐like functionality and support applications in heart repair or drug screening. The relevance for such a two‐tiered screening strategy became particularly obvious when screening two commercially and 24 newly synthesized chalcones. Here, despite a confirmed cardiomyogenesis inducing activity in several chalcones, only one chalcone, 4’‐hydroxychalcone (4’HC; labeled as compound [**1**]), was effective in the generation of cardiomyocytes with the propensity for contractile three‐dimensional EHM formation.

## Results

### Cardiac mesoderm induction

**Figure 1:**
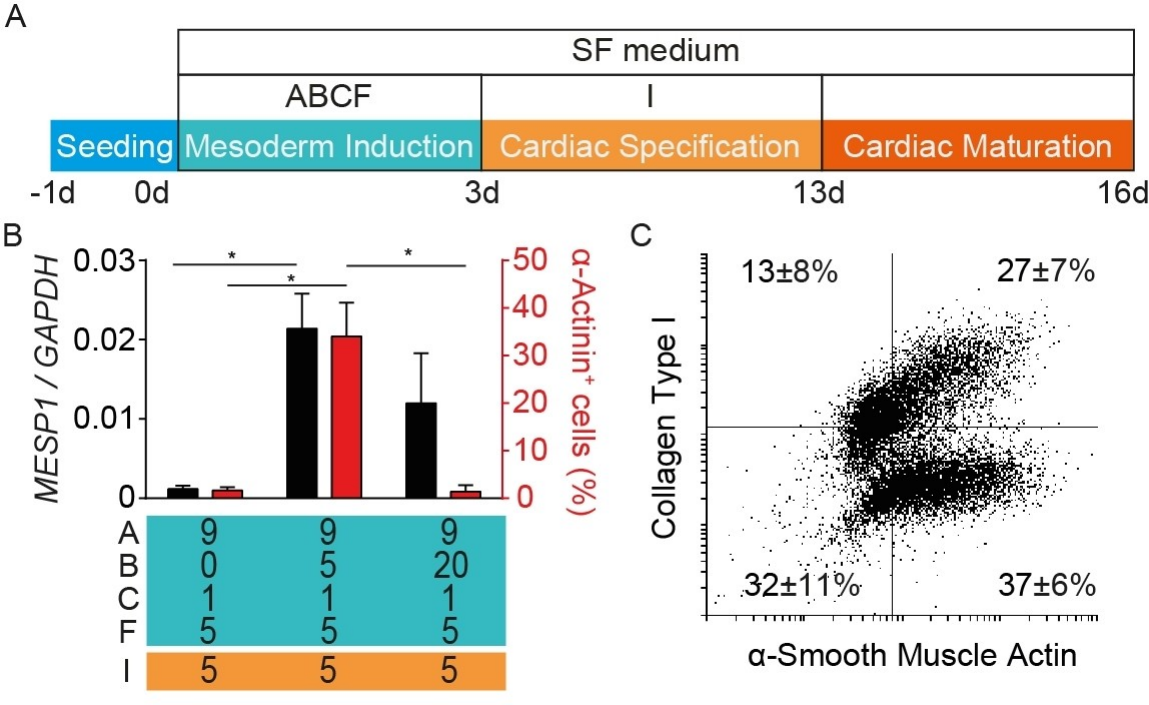
Cardiac mesoderm induction as a function of BMP4 stimulation in 2D culture. (A) Outline of the optimized cardiac differentiation strategy; A: activin‐A, B: BMP4, C: CHIR99201, F: FGF2+, I: IWP4 as recently reported.^[7]^ (B) BMP4 during mesoderm induction (first 3 culture days; *n*=3/group) enhanced *MESP1* transcription (qPCR on culture day 3) and efficiency of cardiac specification/maturation (cytometry for α‐actinin^+^ cells on culture day 16; *n*=3/group); refer to Supporting Information, Figure S2 for gating strategy; concentrations in ng/mL for A, B, and F; in μmol/L for C and I. (C) Flow cytometry (*n*=6 independent experiments) for the identification of fibroblast‐like stromal cells by their expression of α‐smooth muscle actin and collagen type I (alone or in combination depending on activity state).

The importance of BMP4 in the directed cardiac differentiation of hPSC was demonstrated by the omission of BMP4 from our original ABCF+I protocol^[7]^ (Figure 1A: A – activin A at 9 ng/mL; B – BMP4 at 5 ng/mL; C – CHIR99201 at 1 μmol/L; F – FGF2 at 5 ng/mL followed by I – IWP4 at 5 μmol/L for Wnt‐inhibition). Without BMP4, the canonical marker of early pre‐cardiac mesoderm, *MESP1* was not transcriptionally upregulated and accordingly no cardiomyocytes were obtained (Figure 1B, Video S1). The cardiomyogenesis inducing activity of BMP4 was highly concentration dependent with similarly negligible cardiomyocyte yields in the presence of 20 ng/ml BMP4 (Figure 1B). In contrast, the ABCF+I protocol, in which α‐actinin^+^ cell abundance correlated well with cardiomyocyte beating activity, uses 5 ng/mL as a standard cardiomyogenesis inducing concentration (Figure 1B, Video S2). Finally, the 5 ng/mL BMP4 supplemented ABCF+I protocol with no further selection provided cell populations comprising ∼35 % cardiomyocyte, according to flow cytometry of sarcomeric actinin^+^ (Figure 1B) with the remaining cells being primarily fibroblast‐like mesodermal stroma cells (identified as collagen I^+^ and/or α‐smooth muscle actin^+^; Figure 1C), with negligible residual hPSCs (*OCT4 aka POU5F1*), ectodermal (*NEUROD1*), and endodermal (*SOX17*) cell content (Figure S1). Collectively the obtained data confirmed the pivotal concentration‐dependent role of BMP4 in stimulating early cardiac mesoderm induction.

### Chalcones as BMP4 replacements

**Figure 2:**
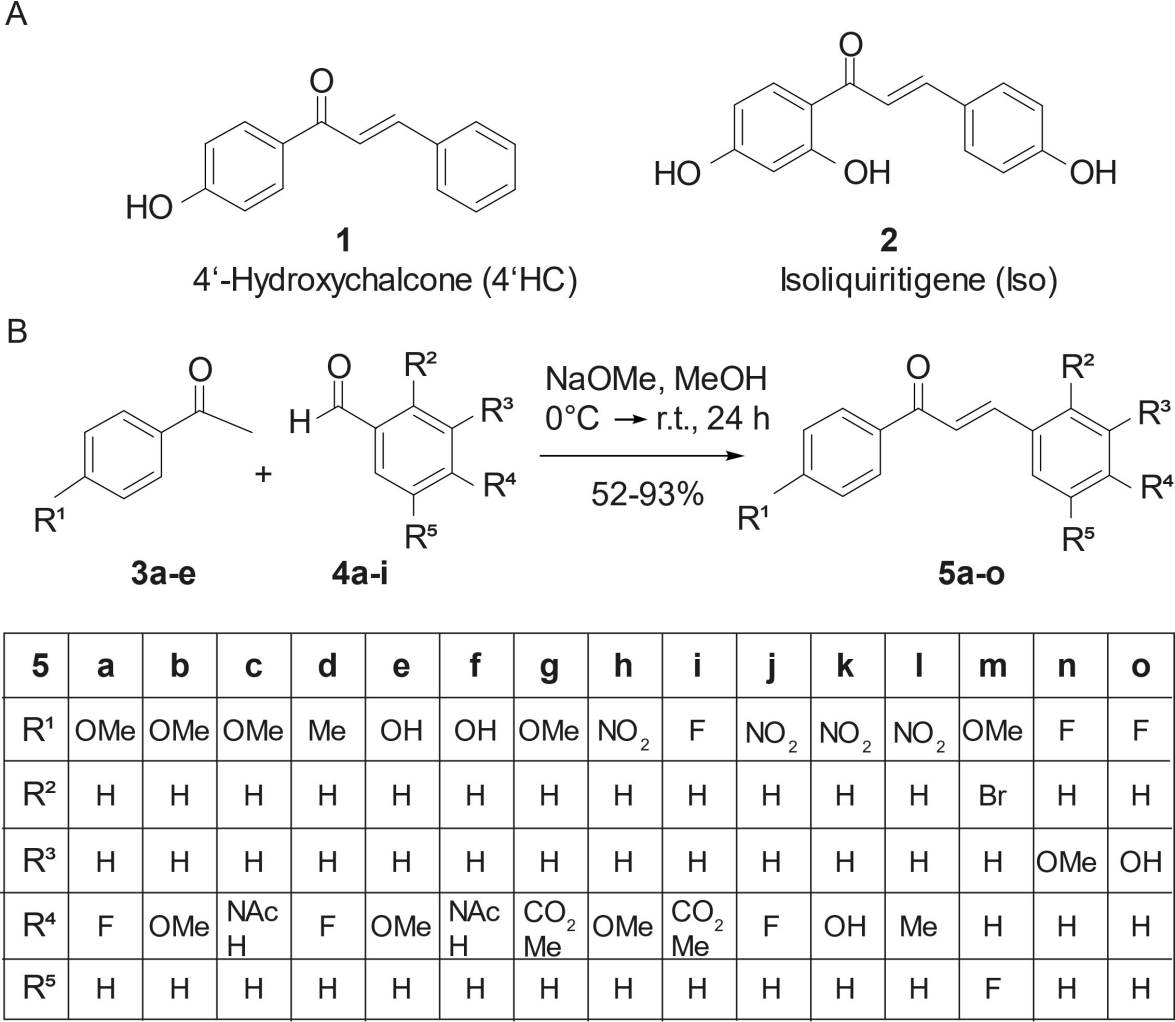
Chemical structures of chalcones used and synthesized in this study. (A) Structure of two commercially available chalcones, 4’‐hydroxychalcone (4’HC, [1]) and Isoliquiritigene (Iso, [2]). (B) Schematic diagram of the chemical synthesis of the 15 novel chalcones and their chemical structures.

**Figure 3:**
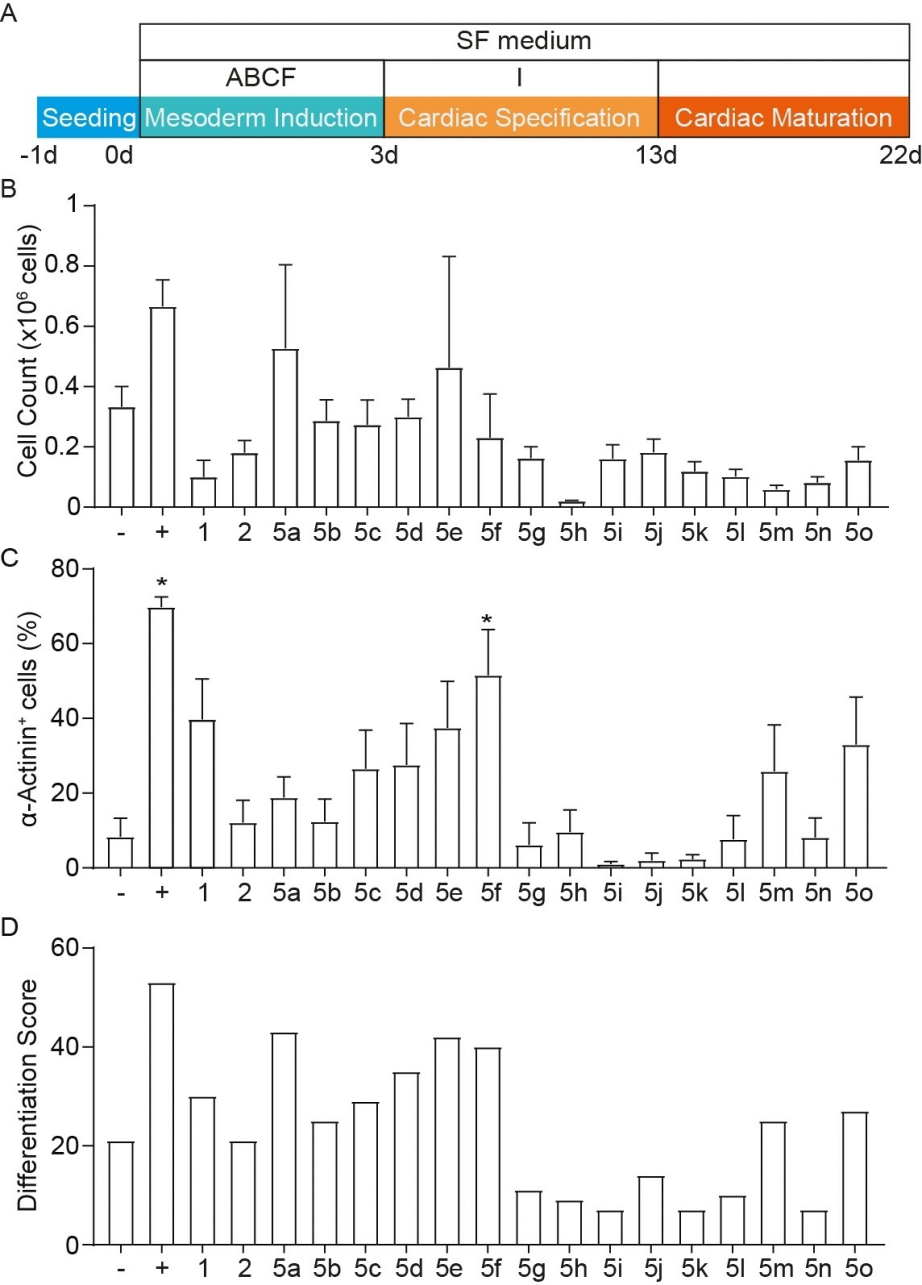
Result of the primary chalcone screen for cardiogenic potential in human PSCs. (A) Outline of the strategy for screening chalcone BMP4 replacements: in the cardiac ABCF+I differentiation protocol, X instead of B indicates the BMP4 replacement. At day 22, (B) cell count (*n*=3–5/group) and (C) cardiomyocyte output measured by α‐actinin expression using flow cytometry (*n*=5–16/group; all from 3 independent experiments) were analyzed. * *P*<0.05 vs. no BMP4 (−) by ANOVA with Dunnett's multiple comparison *post‐hoc* test. (D) Ranking of the different groups based on their DS. (−) No BMP4 (ACF+I); (+) ABCF+I.

We tested 4’HC (compound **1**) and Isoliquiritigene (Iso; designated as compound **2**), which were reported previously for their role in BMP signaling activation during osteoblast differentiation,^[8]^ for a potential mesoderm/cardiomyogenesis inducing activity. We used flow cytometry for α‐actinin assessment at culture day 22 as a robust parameter to quantify cardiomyogenesis and determined that chalcones were most effective at 10 μmol/L, beyond which all cells died due to toxicity (Supporting Information, Figure S3). In an effort to optimize the cardiomyogenesis‐inducing activity, we synthesized 15 novel chalcones (Figure 2) and tested them for their cardiomyogenic potential as BMP4 replacements in the ABCF+I protocol (Figure 3A).

By culture day 22, cardiac cells derived from different conditions were first analyzed for their beating rate and reproducibility (% of cultures with microscopically visible beating cardiomyocytes; Supporting Information, Figure S4A and S4B respectively). To further test the cardiomyocyte derivation efficacy, we evaluated the total cell count (Figure 3B) and cardiomyocyte (CM) content by α‐actinin expression using flow cytometry (Figure 3C). To rank chalcones according to their cardiomyocyte inducing activity, each compound was assigned a numerical rank with “0” being the lowest and “18” the highest attainable values for each of the criteria. For instance, unlike BMP4, which induced the highest cardiac output with 70±3 % of α‐actinin^+^ cells by day 22, compound **5** **i** induced the formation of only 1±0.7 % of α‐actinin^+^ cells (Figure 3C) rendering the latter with a value of “0” and the former with a value of “18”. In case of similar performance, same ranks were assigned and the total score per parameter reduced accordingly. Taking into consideration all criteria and upon summing up the numerical values for each group, a differentiation score (DS) was calculated (Supporting Information, Figure S4C) and plotted (Figure 3D). While BMP4 scored the highest (DS=54), among the chalcone‐treated groups compounds **5** **i**, **5** **k**, and **5** **n** scored the lowest (DS=7 each) and compounds **5** **a**, **5** **e**, and **5** **f** scored the highest (DS=43, 42 and 40 respectively).

**Figure 4:**
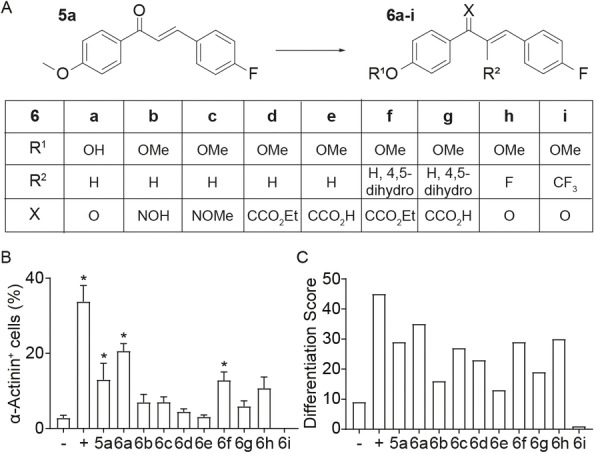
Screen of 4‐flouro‐4’‐methoxychalcone (compound **5** **a**) derivatives for cardiogenic potential in human PSCs. (A) Schematic diagram of the chemical synthesis of nine additional derivatives of compound **5** **a** (top). Screening strategy was similar as indicated in Figure 3A. Analyses were performed on culture day 22 (bottom) to score (B) Cardiomyocyte output measured by flow cytometry detection of α‐actinin^+^ cells (*n*=11–12/group; all from 4 independent experiments). * *P*<0.05 vs. no BMP4 (−) by ANOVA with Dunnett's multiple comparison *post‐hoc* test. (C) Ranking based on the calculated differentiation score (DS). (−) No BMP4 (ACF+I); (+) ABCF+I.

Based on the highest DS and with the aim to further improve its biological activity as BMP4 replacement in the ABXF+I protocol, we selected 4‐fluoro‐4’‐methoxychalcone (compound **5** **a**) as a backbone structure for the synthesis of 9 additional chalcone derivatives (Figure 4A). Using a similar scoring algorithm as applied in the primary screen, 4‐fluoro‐4’‐hydroxychalcone (compound **6** **a**) presented the highest DS among the tested derivatives of compound **5** **a** (Figure 4B and 4C; Supporting Information, Figure S5), while compound **6** **i** induced toxicity and cell death.

**Figure 5:**
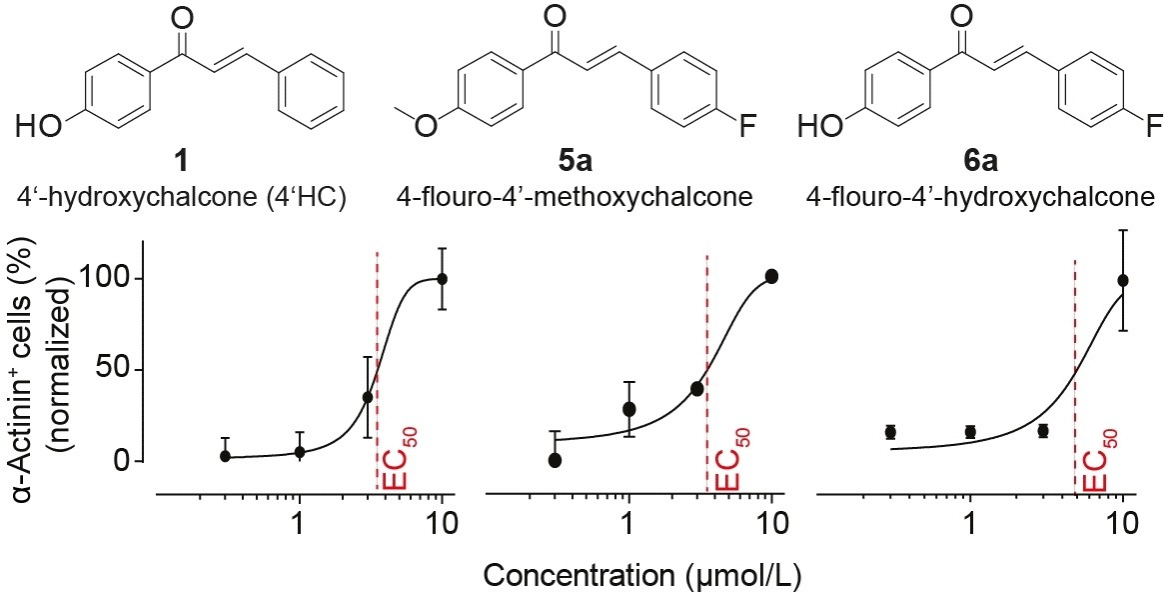
Assessment of chalcone potency in the support of cardiomyocyte derivation. Chemical structure and plot with half‐maximal effect (EC_50_; *n*=3–8/concentration from 3–4 independent experiments) indicated by striped line.

To investigate the potency of the identified lead compounds (i. e. **1**, **5** **a** and **6** **a**), we investigated cardiomyocyte content by day 22 upon the addition of 0, 0.3, 1, 3 and 10 μmol/L of the respective chalcones as BMP4 replacement during cardiac mesoderm induction. Half‐maximally effective concentration (EC_50_) was: 3.5±0.9 μmol/L for **1**, 2.6±0.7 μmol/L for **5** **a** and 4.4±0.5 μmol/L for **6** **a** (Figure 5).

### Molecular and morphological assessment of chalcone‐induced cardiomyocytes

**Figure 6:**
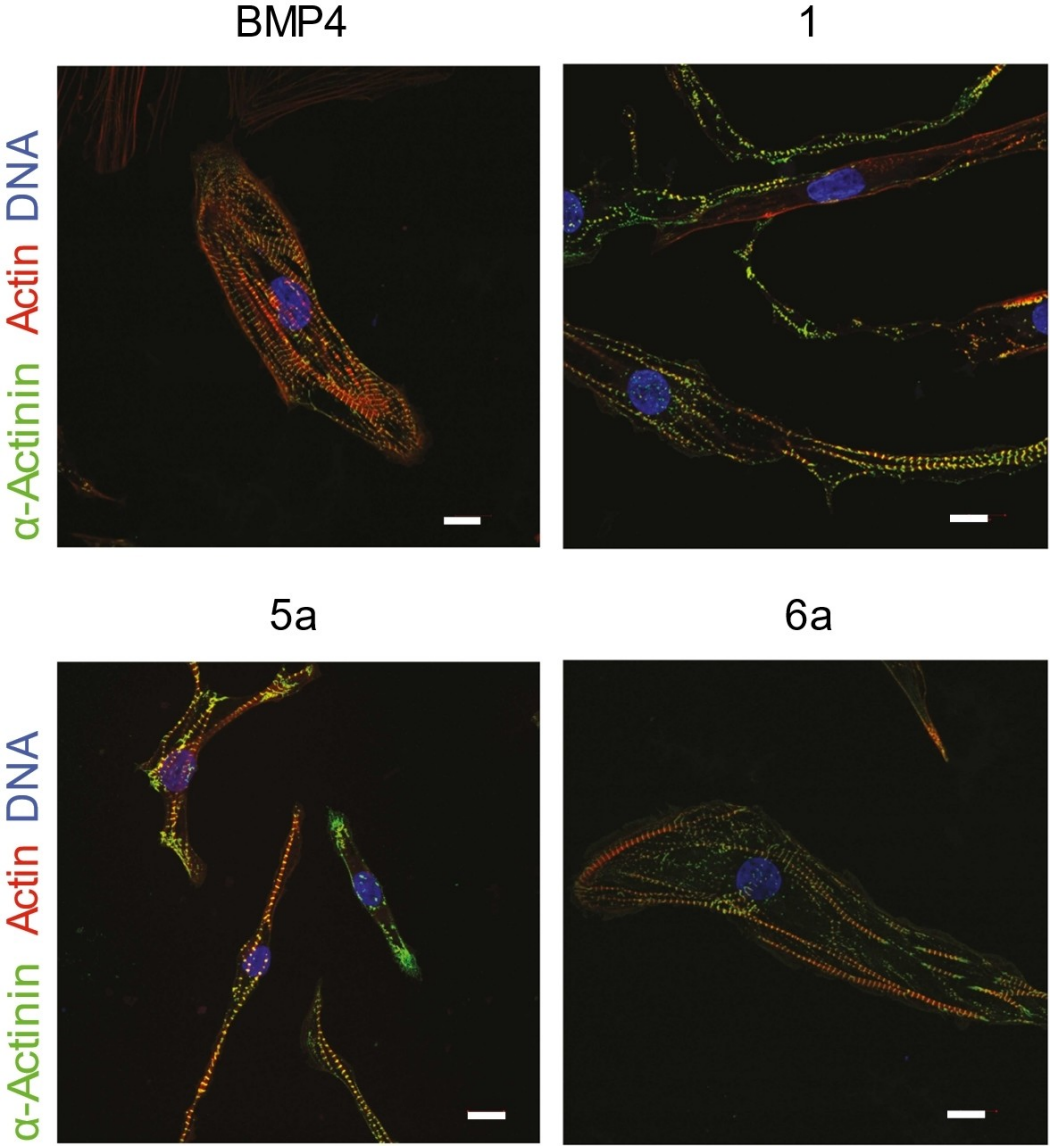
Cardiomyocytes with classical cross‐striation (sarcomere) pattern derived by chalcone replacements of BMP4. Cardiomyocytes derived by the AXCF+I protocol, whereas X was compound **1**, **5** **a** or **6** **a**, stained for α‐actinin (green), actin (red), and DNA (blue) on culture day 22. A cardiomyocyte derived under the standard ABCF+I protocol (includes BMP4) is displayed for comparison. Bars: 20 μm.

By culture day 22, chalcone (**1**, **5** **a** and **6** **a**) treatment‐derived cardiomyocytes presented homogeneous, beating cell clusters (Supporting Information, Videos S3–S5), comprised of cardiomyocytes, identified also by their characteristic sarcomere pattern after labeling of sarcomeric actinin protein (Figure 6). On the molecular level, chalcone treated hPSCs exhibited similar gene transcription profiles as observed in the ABCF+I control protocol; (1) reduced transcription of the pluripotency marker *OCT4* and upregulation of mesodermal markers (*T* and *MIXL1*) along with the expression of the cardiac mesoderm marker *MESP1* peaking at day 3, followed by (2) a cardiac specification phase with the upregulation of early cardiac markers such as *NKX2‐5* and *ISL1* (though peaking at a later stage than the positive control), and (3) a cardiac maturation phase with the upregulation of later cardiac markers such as *MYL2* and *MYL7* by culture day 22 (Supporting Information, Figure S6B).

### Chalcones do not signal via enhanced SMAD phosphorylation

Canonical BMP4 signaling is via a transient induction of SMAD protein phosphorylation. Accordingly, we tested whether the identified cardiomyogenic chalcones would lead to phosphorylation of SMAD1/5/8. Unanticipated and unlike to the activity observed in BMP4, there was no evidence for a transient (after 1 h) increase of SMAD‐phosphorylation. Moreover, SMAD‐phosphorylation was also not enhanced in the BMP4 and chalcone supplemented protocols at the end (72 h) of mesoderm induction (Supporting Information, Figure S7), suggesting collectively that chalcones support mesoderm formation independent of SMAD phosphorylation.

### Only 4’hydroxychalcone supported functional heart muscle formation

**Figure 7:**
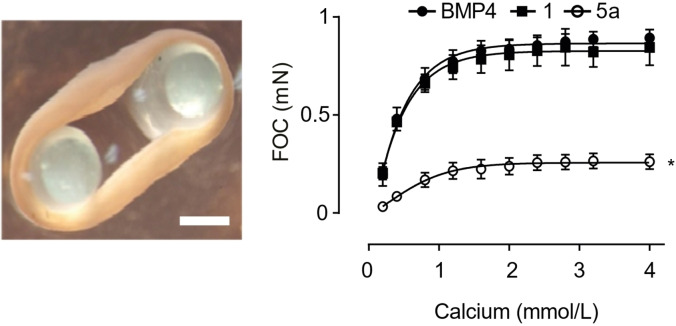
Assessment of heart muscle self‐organization properties. Engineered human myocardium (EHM; image on left; bar: 1 mm) were constructed from purified cardiomyocytes and fibroblasts according to a recently published protocol^[7]^ to investigate the propensity of cardiomyocytes, derived under indicated chalcone support, to self‐organize into contractile heart muscle. Force of contraction (FOC) of EHMs was studied at 1 Hz electrical stimulation under isometric conditions 2 weeks after EHM casting; the graph demonstrates the typical positive inotropic response of EHMs to increasing extracellular calcium concentrations (n=3–5 EHMs/group). * *P*<0.05 vs. BMP4 (ABCF−I protocol) by two‐way ANOVA with Bonferroni's multiple comparison *post‐hoc* test.

For an ultimate test of cardiomyogenesis and the propensity for self‐organization into macroscopically contractile heart muscle, we applied our previously established EHM model.^[7]^ In the EHM assay, human cardiomyocytes are mixed with human fibroblasts and subjected to defined culture conditions to form macroscale contractile human heart muscle. Note that we tested cardiomyocytes derived from the compound **1** and **5** **a** supplemented AXCF+I protocol only, because of the less favorable EC_50_ in compound **6** **a** (Figure 5). Interestingly, cardiomyocytes under compound **1**, but not compound **5** **a** supplementation were capable of self‐organization into EHM (Figure 7). This finding was surprising and supports the need for deep tissue phenotyping beyond classical cardiomyocyte counts (typically performed by flow cytometry and analysis of beating activity) to identify compounds with *bona fide* cardiomyogenesis inducing activity.

## Discussion

Lessons from embryonic development demonstrated the importance of the spatio‐temporal activation/inhibition of key signaling pathways (i. e., BMP, Activin, Nodal and Wnt signaling pathways) to direct stem cells towards the cardiac lineage.[^4c^, ^4d^, ^10^] We have previously reported the utility of the sequential addition of growth factors (Activin‐A, BMP4, CHIR, and FGF2 [ABCF] for mesoderm induction) and a small molecule Wnt‐signaling inhibitor (IWP4 for cardiac specification [+I]) in the derivation of cardiomyocytes from several hPSC lines with similar efficiency (ABCF+I protocol).^[7]^ Similar results can be obtained with alternative small molecule protocols.^[11]^ However, across species applications, which are important for example for bridging studies from non‐human primates to human, showed suboptimal results under small molecule Wnt‐modulation (the GiWi protocol) and versatile applicability of the ABCF+I protocol.^[12]^

To reduce the dependency on growth factors such as BMP4 with known variability in biological activity, we focused our study on the identification of small molecule BMP4 replacements. After the identification of chalcones as activators of BMP signaling by a literature screen,^[8]^ we tested two commercially available chalcones and identify 4’‐hydroxychalcone (**1**) as similarly effective as BMP4. Two derivatives, i. e., compound **5** **a** (4‐fluoro‐4’‐methoxychalcone) and **6** **a** (4‐fluoro‐4’‐hydroxychalcone), showed similar activity in 2D cultures with however different potency (EC_50_: **5** **a**<**6** **a**). Despite apparent similarities in cardiomyogenesis induction in **1** and **5** **a** it was obvious that the quality of the resulting cardiomyocytes differed, i. e., only under replacement of BMP4 by compound **1** cardiomyocytes with the propensity to self‐organize into strongly contracting EHM could be obtained. These findings collectively demonstrate the importance of a sequential 2D (higher throughput) and 3D (lower throughput, but tissue level relevance) phenotyping strategy for the identification of cardiomyogenesis supporting new molecular entities.

We acknowledge that further optimization of the chalcone chemistry may be desirable, but note that compound **1** (4’‐hydroxychalcone) is equally effective as BMP4 in the ABCF−I protocol as to the derivation of heart muscle formation competent cardiomyocytes. Moreover, it is likely that other chalcone derivatives may also be effective at carefully defined concentrations. In this context, it is important to note that also the BMP4 effects are highly concentration dependent with optimal activity as to the induction of cardiomyogenesis in a narrow concentration range (optimal effect at 5 ng/mL – failure at 0 and 20 ng/mL).

## Conclusion

With the identification of a small molecular BMP4 replacement, a first step is taken to chemically define the ABCF+I protocol for effective cardiomyogenesis induction. Screening for replacements for Activin‐A and FGF2 as well as other cardiomyogenesis or heart function modifying compounds will follow a similar two‐tiered strategy with contractile performance at the tissue level being the ultimate phenotypic measure. Before clinical use a deeper characterization of the ultimately anticipated chemically defined ABCF+I inspired protocol will be required.

## Affiliations

From the Institute of Pharmacology and Toxicology, University Medical Center Göttingen, Germany (F. R., T. U. E., J. E. H., B. I. S., B. F., W.‐H. Z.); German Center for Cardiovascular Research (DZHK), partner site Göttingen, Germany (F. R., T. E., J. E. H., B. I. S., B. F., W.‐H. Z.); Institute of Organic and Biomolecular Chemistry, Georg‐August University Göttingen, Germany (T. A. K., R. G., L. F. T.). The current address of Farah S. Raad is Lab of Special Cellular Toxicology, Non‐clinical Drug Safety, Boehringer Ingelheim International GmbH, Biberach Site, Germany. The current address of Taukeer A. Khan is Department of NanoBiophotonics, Max Planck Institute for Biophysical Chemistry, Göttingen, Germany. The current address of Tilman U. Esser is Department of Nephropathology, Institute of Pathology, Friedrich‐Alexander‐Universität Erlangen‐Nürnberg (FAU), Germany. The current address of James E. Hudson is QIMR Berghofer Medical Research Institute, Royal Brisbane Hospital, Brisbane, Australia. The current of Bhakti I. Seth is Max Delbrueck Center for Molecular Medicine, Berlin, Germany. The current address of Buntaro Fujita is Department of Cardiac and Thoracic Vascular Surgery, University Hospital of Schleswig‐Holstein, Luebeck Campus, Germany. The current address of Ravi Gandamala is Sai life Sciences, India.

## Supporting information

As a service to our authors and readers, this journal provides supporting information supplied by the authors. Such materials are peer reviewed and may be re‐organized for online delivery, but are not copy‐edited or typeset. Technical support issues arising from supporting information (other than missing files) should be addressed to the authors.

Supporting Information

Supporting Information

Supporting Information

Supporting Information

Supporting Information

Supporting Information
