# Supplementary material for: Chalcone‐Supported Cardiac Mesoderm Induction in Human Pluripotent Stem Cells for Heart Muscle Engineering
Source: ChemMedChem. 2021 Aug 31;16(21):3300–5. doi: 10.1002/cmdc.202100222 (PMC8597156; doi:10.1002/cmdc.202100222)
Supplement: Supplementary file 1 — Supporting Information [file CMDC-16-3300-s001.pdf]

ChemMedChem

Supporting Information

Chalcone-Supported Cardiac Mesoderm Induction in Human Pluripotent Stem Cells for Heart Muscle Engineering

Farah S. Raad, Taukeer A. Khan, Tilman U. Esser, James E. Hudson, Bhakti Irene Seth, Buntaro Fujita, Ravi Gandamala, Lutz F. Tietze, and Wolfram Zimmermann*

Supporting Information

Supplementary Figures 1-7	Pages 2-8
Supplementary Tables 1-2	Pages 9-10
Legends for Supplementary Videos 1-5	Page 11
Experimental Section	Pages 12-15
Synthesis Schemes	Pages 16-33
References	Page 34

Supplementary Figures

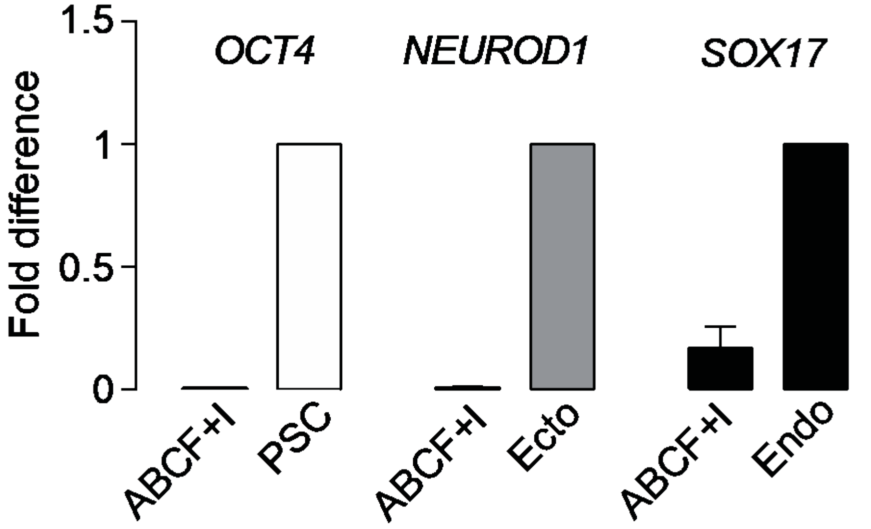

Figure S 1: Negligible transcription of ectodermal and endodermal marker genes in response to the ABCF+I protocol. qPCR screen for residual PSCs (OCT4), ectodermal cells (NEUROD1), and endodermal cells (SOX17; n = 3/group) in ABCF+I-derived heart muscle cells; undifferentiated HES2 and derivatives (ectodermal [Ecto] and endodermal [Endo] induction) served as reference material.

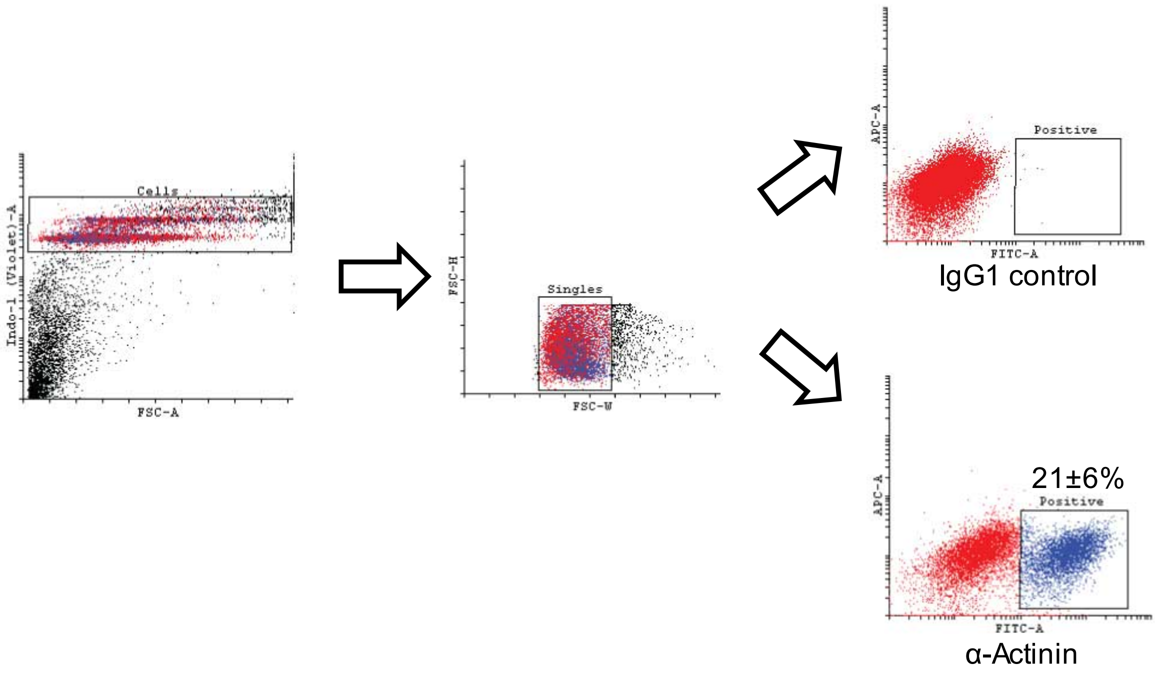

Figure S 2: Gating strategy for the assessment of cardiomyocyte derivation from hPSC by flow cytometry. Intact cells were gated on the DNA label "Hoechst 33342" fluorescence intensity (FI; left panel), then forward scatter width and height (FSC-W; FSC-H) to exclude doublets. Cardiomyocytes were identified by a positive signal after antibody labeling of sarcomeric α-actinin (ACTN2). Representative plots for IgG_1 isotype control and α-actinin on the right (data from $n = 6$ experiments); FSC: forward scatter; FSC-A: FSC-signal area; FSC-H: FSC-signal height; FSC-W: FSC-signal width.

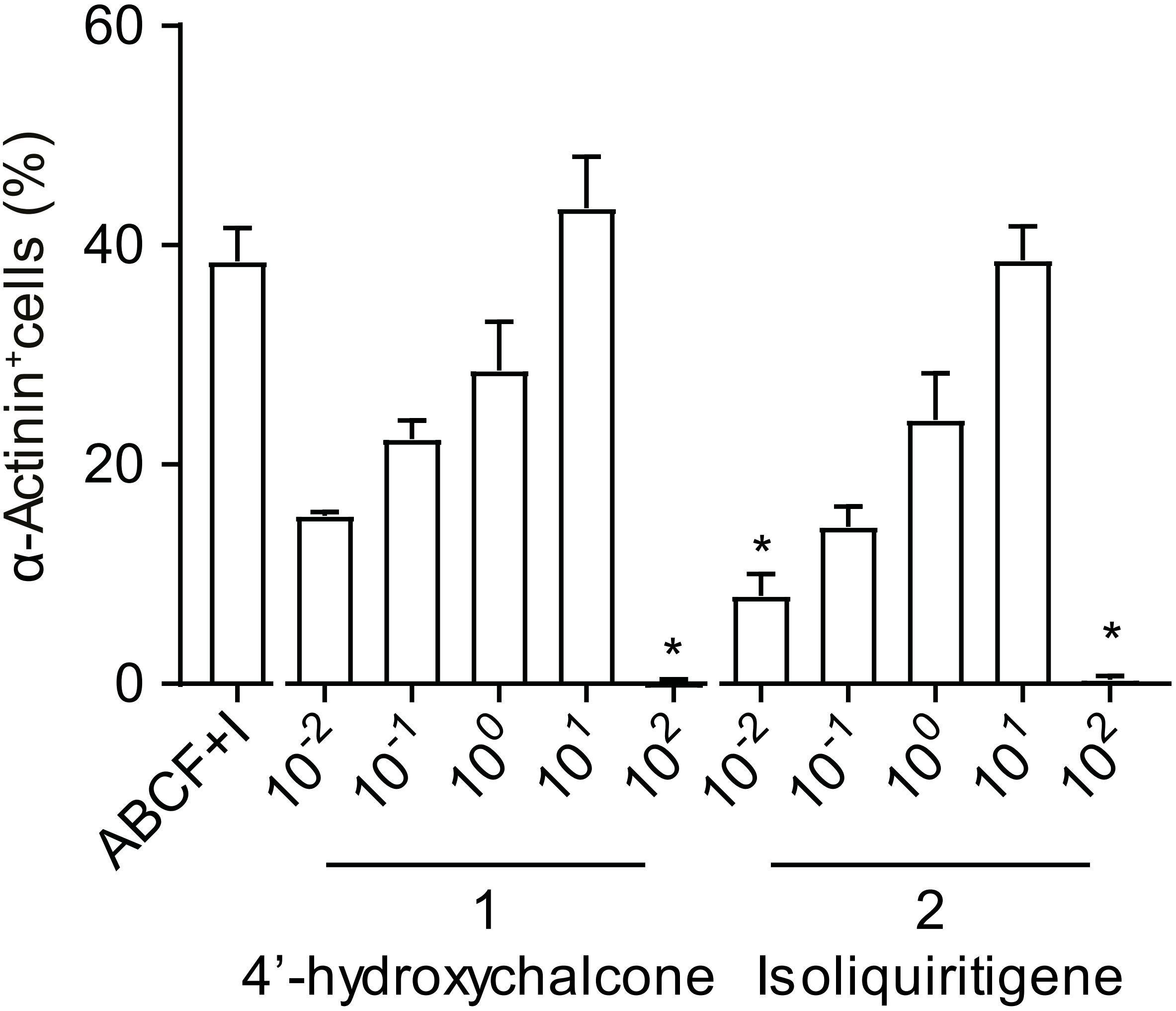

Figure S 3: Chalcone concentration dependent effects on cardiomyogenesis. Flow cytometry analysis of cardiomyocyte content (% α-actinin positive cells on culture day 22) after exposure of hPSCs to the AXCF+I protocol, whereas X refers to the commercially available chalcones 4'-hydroxychalcone (**1**) and Isoliquiritigene (**2**) tested at the indicated concentrations (in µmol/L; n = 3-17 biological replicates/concentration). * $P < 0.05$ vs. ABCF+I by ANOVA with Dunnett's multiple comparison *post-hoc* test.

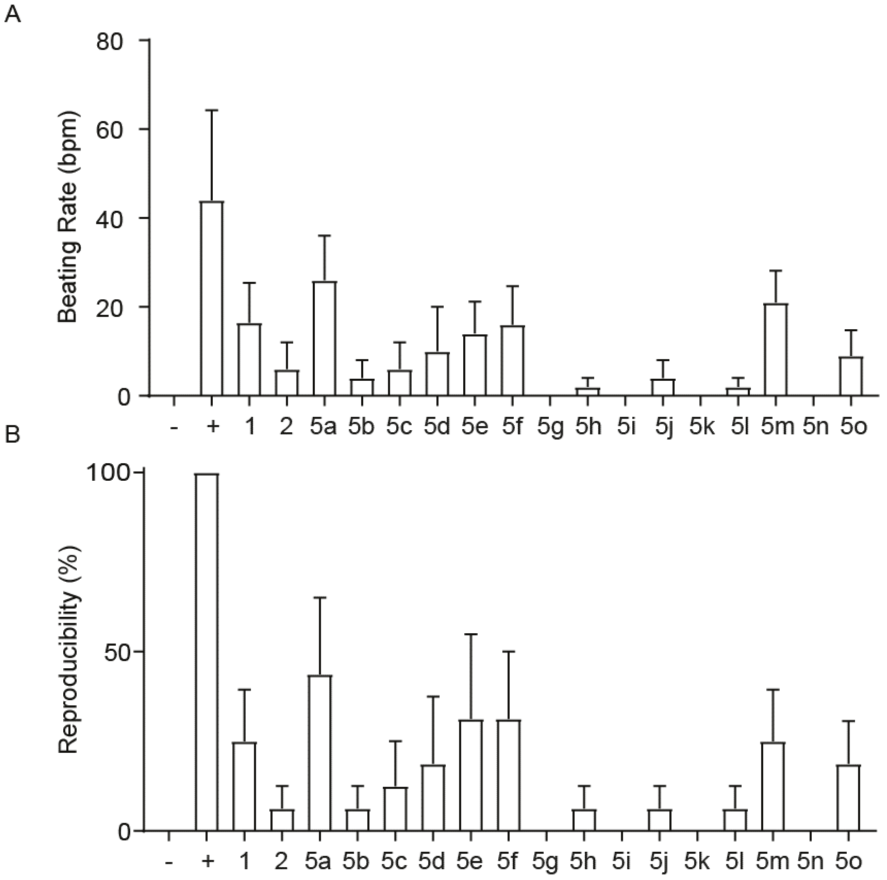

C

Sample	BR	Repro	Cell count	CM	DS
no BMP4 (-)	0	0	15	6	21
BMP4 (+)	11	7	18	18	54
1	8	4	3	16	31
2	3	1	9	8	21
5a	10	6	17	10	43
5b	2	1	13	9	25
5c	3	2	12	12	29
5d	5	3	14	13	35
5e	6	5	16	15	42
5f	7	5	11	17	40
5g	0	0	8	3	11
5h	1	1	0	7	9
5i	0	0	7	0	7
5j	2	1	10	1	14
5k	0	0	5	2	7
5l	1	1	4	4	10
5m	9	4	1	11	25
5n	0	0	2	5	7
5o	4	3	6	14	27

Figure S 4: Primary screening results. Assessment of **(A)** Beating Rate (BR) of the derived cardiomyocytes (n = 3-4 /group) and **(B)** Reproducibility (Repro; % of cultures with visibly beating cardiomyocytes - 4 wells analyzed/differentiation run; n = 4 differentiation runs/group) at culture day 22 following the screen of 2 commercially available and 15 newly synthesized chalcones. **(C)** Summary of numerical values assigned for each analyzed parameter; CM: cardiomyocytes content in total cell count; DS: differentiation score, summation of the individual scores for each analyzed parameter. - : no BMP4 (ACF+I); + : ABCF+I

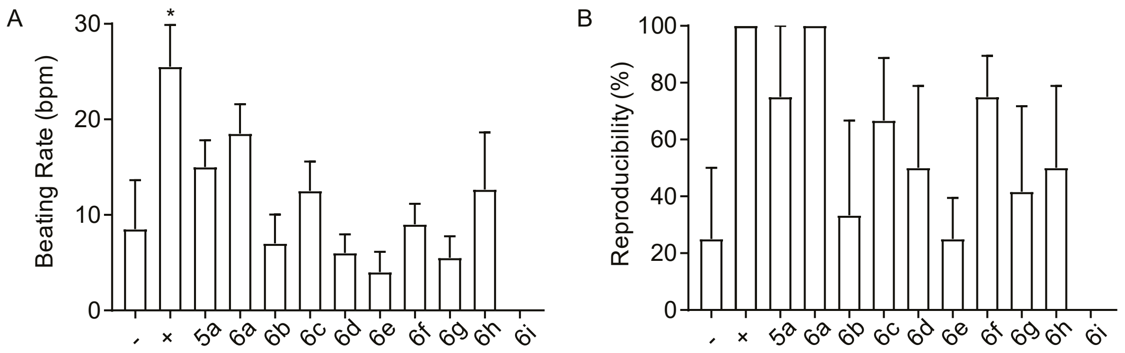

Figure S 5: Screen of 4-fluoro-4'-methoxychalcone (compound 5a) derivatives. Analyses were performed similarly as described for the primary screen of the first round of commercial and newly synthesized chalcones. **(A)** Beating Rate (n = 12/group) and **(B)** Reproducibility (% of cultures with visibly beating cardiomyocytes – 4 wells analyzed/differentiation run; n = 3 differentiation runs/group). * $P < 0.05$ vs. no BMP4 (-) by ANOVA with Dunnett's multiple comparison *post-hoc* test. - : no BMP4 (ACF+I); + : ABCF+I

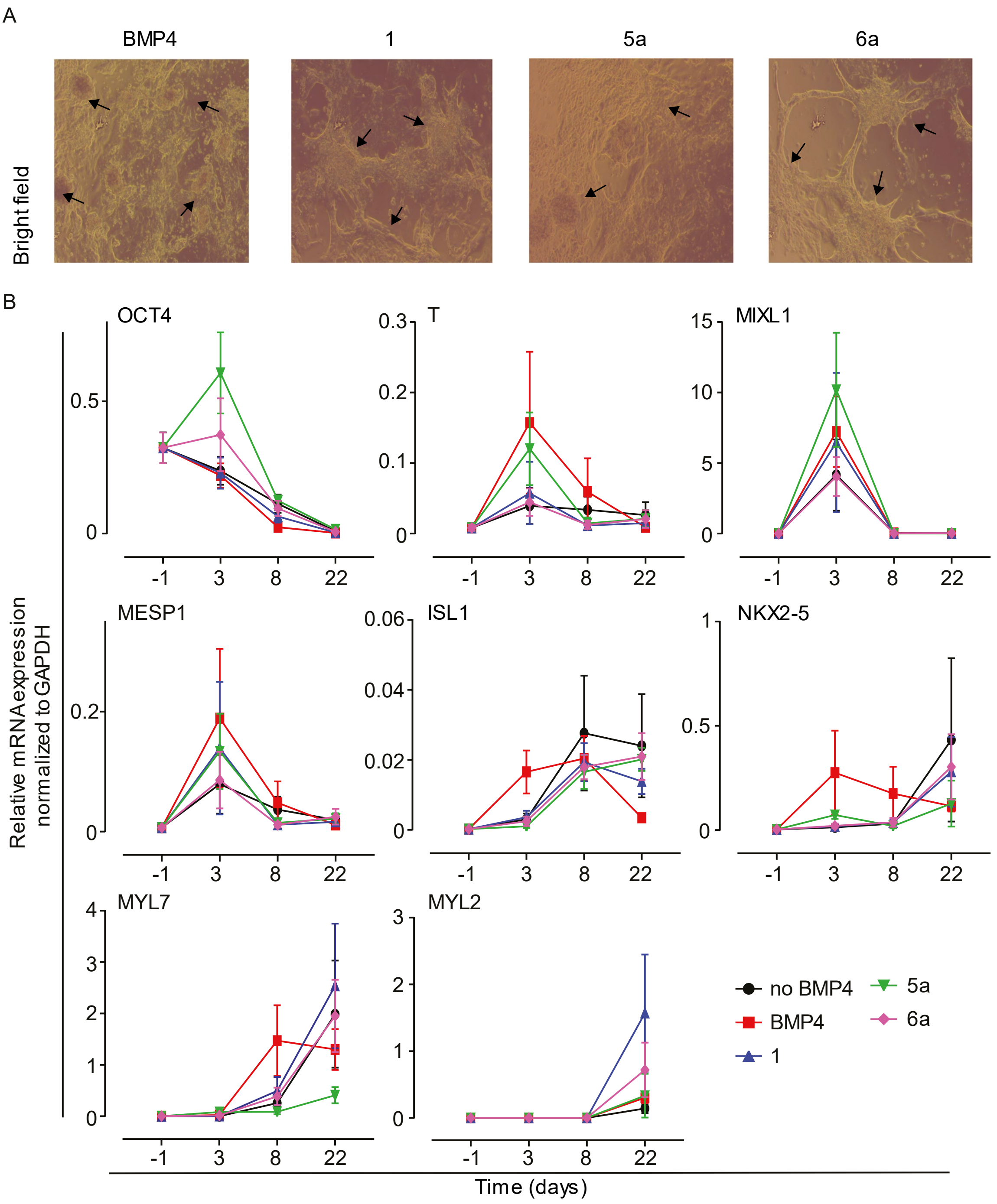

Figure S 6: Morphological and transcript profiling of cardiomyocytes derived by the 4'-hydroxychalcone (1) as well as compound 5a and 6a supplemented AXCF+I protocol. (A) Brightfield images with arrows indicating beating cardiomyocytes clusters; refer also to **Supplementary Videos**. **(B)** Relative gene expression of pluripotent marker *OCT4*, early mesodermal markers *T* and *MIXL1*, cardiac mesodermal marker *MESP1*, early cardiac markers *ISL1* and *NKX2-5* and late cardiac markers *MYL7* and *MYL2* during differentiation using qPCR. All data were normalized to *GAPDH* transcript abundance (*n*= 3-5 samples/time point).

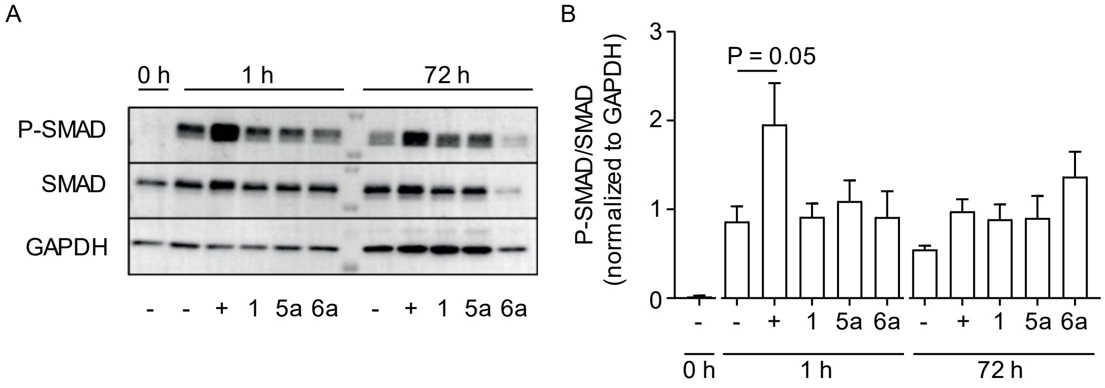

Figure S 7: **No evidence for chalcone signaling via the SMAD pathway.** (**A**) Western blot analyses of P-SMAD1/5 and total SMAD1 in cell lysates at 0, 1 (after medium change), and 72 h post-treatment with BMP4 (5 ng/mL), **1**, **5a**, and **6a** (all at 10 µmol/L). GAPDH was used as loading control (left). (**B**) Summary of the Western blot data showing relative phosphorylated protein normalized to GAPDH (right; *n*=3 samples/group). - : no BMP4 (ACF+I); + : ABCF+I

Table S1. List of Antibodies used in this study.

(FC – Flow Cytometry, IF – Immunofluorescence, WB – Western Blot)

Antibody/Stain	Company	Cat No.	Target / Use	Dilution
IgG_1	R&D Systems	MAB002	Control	1:1,000 FC<br>1:100 IF
α-actinin (mouse IgG)	Sigma	A7811	Cardiomyocyte	1:4,000 FC<br>1:1,000 IF
α-smooth muscle actin (mouse IgG)	Sigma	A2547	Stromal Cell	1:4,000 FC<br>1:400 IF
Collagen I (rabbit IgG)	Abcam	Ab34710	Stromal Cell	1:2,000 FC<br>1:500 IF
Goat anti-mouse Alexafluor 488	Invitrogen	A-11001	Secondary Antibody	1:1,000 FC<br>1:400 IF
Goat anti-rabbit Alexafluor 546	Invitrogen	A-11010	Secondary Antibody	1:1,000 FC<br>1:400 IF
Phospho-SMAD1/5 (Rabbit)	Cell Signaling	9516S	Phosphorylated SMAD1/5 (60 kDa)	1:500 WB
SMAD1 (Rabbit)	Cell Signaling	9743S	Total SMAD1 protein (60 kDa)	1:1,000 WB
Anti-mouse-IgG HRP conjugated	Dako	P0161	Secondary Antibody	1:10,000 WB
Anti-rabbit-IgG HRP conjugated	Dako	0448	Secondary Antibody	1:5,000 WB

Table S2. List of Primers used in this study

Gene	F	R	Purpose	Size (bp)	Acc #
OCT4	CAGTGCCCGAAACCCACAC	GGAGACCCAGCAGCCTCAAA	qPCR	161	NM_002701<br>NM_203289<br>NM_001173531
T	TCAGCAAAGTCAAGCTCACCA	CCCCAACTCTCACTATGTGGATT	qPCR	102	NM_003181
MIXL1	CCGAGTCCAGGATCCAGGTA	CTCTGACGCCGAGACTTGG	qPCR	58	NM_031944
NKX2-5	ACAACTTCGTGAACTTCGGCG	GTGGACACTCCCGAGTTGCTCT	qPCR	82	NM_001166175.1<br>NM_001166176.1<br>NM_004387.3
MESP1	CCACCGTCCCCGCTCCTTCC	CGGTGCTCACAGAGACGGCG	qPCR	130	NM_0018670
ISL1	CGCCTTGCAGAGTGACATAG	GGACTGGCTACCATGCTGTT	qPCR	147	NM_002202.2
MYL7 (MLC2a)	CAGCGGCAAAGGGGTGGTGAAC	GGTCCATGGGTGTCAGGGCGAA	qPCR	113	NM_021223.2
MYL2 (MLC2v)	GGCGCCAACTCCAACGTGTT	ACGTTCACTCGCCCAAGGGC	qPCR	149	NM_000432.3
NEUROD1	AGCCACGGATCAATCTTCTC	GCGTGCCTCTAATCATGAAA	qPCR	143	NM_002500.3
SOX17	AGGAAATCCTCAGACTCCTGGGTT	CCCAAACTGTTCAAGTGGCAGACA	qPCR	111	NM_022454.3
GAPDH	CCTCAAGATCATCAGCAATGCC	ATGTTCTGGAGAGCCCCGC	qPCR	189	NM_002046.3

Video Legends:

Video S1: HES2-cardiomyocytes derived in the absence of BMP4 (ACF+I protocol). Negative control: No spontaneous beating of cardiomyocytes on culture day 22.

Video S2: HES2-cardiomyocytes derived in the presence of BMP4 (ABCF+I protocol). Positive control: spontaneous coordinated beating of cardiomyocytes on culture day 22.

Video S3: HES2-cardiomyocytes derived from the AXCF+I protocol whereas X was 4'-hydroxychalcone (compound [cpd] 1). Beating of cardiomyocytes was recorded on culture day 22.

Video S4: HES2-cardiomyocytes derived from the AXCF+I protocol whereas X was 4-fluoro-4'-methoxychalcone (compound [cpd] 5a). Beating of cardiomyocytes was recorded on culture day 22.

Video S5: HES2-cardiomyocytes derived from the AXCF+I protocol whereas X was 4-fluoro-4'-hydroxychalcone (compound [cpd] 6a). Beating of cardiomyocytes was recorded on culture day 22.

Experimental Section

Ethics. The import and use of human embryonic stem cells (HES2-ROSA26-RFP [HES2])[1]; was approved by the Central Ethics Committee for Stem Cell Research according to the German Stem Cell Act (Az 1710-79-1-4-16).

PSC culture. HES2 were confirmed to be karyotypically normal, mycoplasma free and pluripotent using in vivo teratoma studies, immunofluorescence staining and flow cytometry. Feeder-cell free culture adapted HES2 were seeded at 25,000 cells/cm^2 on Matrigel^{TM} (1:30 in phosphate-buffered saline [PBS]; BD Biosciences)-coated T75 flasks in TeSR-E8 medium (E8; Stemcell Technologies), supplemented with antibiotics (100 IU/ml penicillin and 100 µg/ml streptomycin; Gibco). After 48 h, HES2 were passaged by dispersion in TrypLE (Gibco) for 3 min at 37 °C and seeded at 50,000 cells/cm^2 on Matrigel™ coated 24-well plates (culture day -1).

Standard cardiac differentiation protocol. We applied the ABCF+I cardiac differentiation protocol (**Figure 1A**),[2] which we introduced previously with robust cardiomyogenesis-inducing activity in all so far tested hPSC lines, including several human embryonic and induced pluripotent stem cell lines. In brief, mesoderm induction was for 3 days with ABCF: Activin A (9 ng/ml), BMP4 (5 ng/ml), CHIR99021 (1 µmol/L) and FGF2 (5 ng/ml) in serum-free (SF) medium (RPMI 1640 supplemented with 2% B27 with insulin, 1 mmol/L sodium pyruvate [all Gibco] and 200 µmol/L L-ascorbic acid 2 phosphate sesquimagnesium salt hydrate [ASC; Sigma] and antibiotics). This was followed by cardiac specification by supplementing SF-medium with IWP4 (5 µmol/L) for 10 days beyond which cultures were maintained in SF medium until day 22.

Screening for a small molecular BMP4 replacement. We tested commercially available (4'hydroxychalcone [Cat#: CDS003892] and Isoliquiritigene [Cat#: I3766]; both from Sigma-Aldrich) and 24 newly synthesized small molecular weight compounds of the chalcone family (for details see **Synthesis Schemes** below) as BMP4 replacements (AXCF+I protocol). Candidate compounds were ranked according to their cardiomyogenesis inducing activity using a combined differentiation score (DS) derived from an assessment of multiple parameters: (1) reproducibility of cardiomyocyte derivation, (2) cardiomyocyte beating rate, (3) total cell number as well as (4) percentage and (5) total quantity of α-actinin positive cardiomyocytes at culture day 22. More detailed analyses were performed with the most effective compounds as indicated.

Compound Syntheses. A mechanically stirred solution of acetophenone (1 mmol/L) in dry MeOH (3 mL) was treated with a solution of NaOMe (5.4 M in MeOH, 5 mmol/L) at 0 °C and stirring was continued for 1 h at room temperature. Then a solution of aldehyde (1 mmol/L) in MeOH (5 mL) was added dropwise and the reaction mixture was stirred for additional 24 h. The solution was treated with H_2O (20 mL), acidified to pH = 1 with HCl (5 mL of a 10% aq. solution), stirred for 10 min and extracted with EtOAc (4 × 15 mL). The combined organic layers were washed with brine (20 mL), dried over $MgSO_4$ and concentrated under reduced pressure. Purification by flash column chromatography (EtOAc:PE = 1:9→1:2) yielded the target compounds 5a-o as indicated in **Figure 2B**; synthesis of compounds 5e[3], j[4], k[5], n[6], and o[5a, 7] were described previously. A detailed compound structure analyses of all compounds and the synthesis procedures for compounds 6 a-i (summarized also in **Figure 4A**) are listed below.

Chromatography. Thin-layer chromatography was performed on silica gel plates (TLC Silica gel 60 F_{254}; Merck). Column chromatography was performed with silica gel (Geduran® Si60, *Ø* = 32–64 µm; Merck).

*NMR spectroscopy***.** NMR spectra were recorded on *Varian* "Mercury-300", "Unity-300", "Inova-500" and on a "AMX-300" spectrometer from *Bruker*. Chemical shifts are given in ppm relative to tetramethylsilane (TMS) and coupling constants *J* in Hertz. Solvent signals serve as reference and the chemical shifts converted to the TMS scale ($CHCl_3$: δ H = 7.24 ppm, δ C = 77.16 ppm). Multiplicities of first order signals are assigned as: s (singlet), sbr (broad singlet), d (doublet), t (triplet), q (quartet), dd (doublet of doublets). Signals of higher order are declared as m (multiplet).

IR spectroscopy. IR spectra were recorded with a FT/IR-4100 spectrometer from *JASCO* (substances were applied neat on an ATR unit).

UV spectroscopy. UV spectra were recorded on a *JASCO* V-630 spectrometer.

Mass spectrometry. ESI-MS and ESI-HRMS spectra were recorded on a "Apex IV" spectrometer from *Bruker Daltronik.*

Cell dissociation for cell counting and flow cytometry. On culture day 22, cells were washed in PBS and dissociated by incubation in dissociation reagent (Accutase [Gibco] supplemented with 0.025% Trypsin [Gibco] and 20 µg/ml DNaseI [Merck]) for 30-45 min. The cells were

pelleted by centrifugation (300 g, 3 min) and fixed with either 4% formaldehyde (FA) for immunofluorescence staining or 70% ice cold ethanol for flow cytometry analysis.

Flow cytometry. Depending on the protein of interest, cells were incubated with primary antibodies (**Table S1**) raised against sarcomeric actinin (Sigma), smooth muscle actin (Sigma) and collagen type I (Abcam) in blocking buffer (containing 5% FBS, 1% bovine serum albumin [BSA], 0.5% Triton X-100 in PBS) for 45 min followed by appropriate secondary antibodies (**Table S1**) in blocking buffer and Hoechst 33342 (10 µg/mL) for 30 min at 4°C. Flow cytometry was performed with a BD LSRII SORP instrument (BD Biosciences). IgG isotype controls (R&D Systems) were used to determine non-specific staining. The gating strategy is displayed in **Figure S2**.

Immunofluorescence staining. Cells were plated on Matrigel™-coated glass coverslips for 24 h in SF-medium before fixation in 4% FA. After blocking for 30 minutes in blocking buffer, primary antibodies (**Table S1**) were added for 90 min followed by secondary antibodies (**Table S1**) and Hoechst 33342 (10 µg/mL) for 60 min at room temperature. Coverslips were mounted onto glass slides (SuperFrost Plus, Thermo Fisher Scientific) using Fluoromount (Dako) and imaged using a Zeiss 710 NLO confocal microscope or a Zeiss AxioImager.M2 fluorescence microscope.

Polymerase chain reaction. Cells were harvested and RNA extraction was performed using Trizol™ following manufacturer's instructions (Ambion). RNA (1 µg) was treated with DNAse (Roche) followed by cDNA synthesis using a High Capacity cDNA Reverse Transcription Kit (Applied Biosystems). Quantitative PCR (qPCR) was performed using the Fast SYBR Green Master Mix (Applied Biosystems) in a 384-well format AB7900 HT (Applied Biosystems). Gene expression analyses were performed according to the standard curve method.[8] *GAPDH* transcript abundance was used for normalization. Primer details are given in **Table S2**.

Immunoblotting. Cells were lysed using CytoBuster reagent (Merck) supplemented with phosphatase- and proteinase-inhibitor cocktail (Roche). Protein concentrations were determined using a Bradford assay. Protein samples were separated by SDS-PAGE and transferred onto polyvinylidene difluoride (PVDF, GE lifesciences) membranes. Membranes were then suspended in blocking buffer containing 5% milk in TRIS-buffered saline with Tween (TBST; 0.02 mol/L TRIS-hydrochloride, 0.17 mol/L NaCl and 0.1% Tween-20 in H_2O) for 1 hour, incubated with primary antibody (**Table S1**; in 5% BSA in TBST) overnight and with secondary antibody at room temperature for 1 hour (**Table S1**). The proteins of interest

were detected using SuperSignal HRP Substrate (Thermo Fisher Scientific) in a ChemiDoc MP Imaging system (Bio-Rad). Signals were normalized to the expression of GAPDH (Zytomed Systems).

EHM casting. EHM were reconstituted according to a previously described protocol.[2] Prior to casting and to enhance cardiomyocyte purity, differentiated cells were first subjected to metabolic selection[9] in RPMI (without Glucose) with 0.1 mmol/L 2-mercaptoethanol, antibiotics (all Gibco) and 2.2 mmol/L sodium lactate (Sigma) for 4 days, followed by a recovery and further maturation phase again in SF medium until culture day 22. This increased cardiomyocyte population to 66±3% for **1** and 24±4% for **5a** (data collected from *n*=6-10 experiments). HES2-derived cardiomyocytes ($1.01x10^6$ CMs/EHM) and human foreskin fibroblasts (ATCC; $0.44x10^6$ cells/EHM) were suspended in a mixture of bovine collagen (LLC Collagen Solutions, 0.4 mg/EHM) and serum-free medium (2x RPMI, 8% B27 without insulin, 200 U/ml penicillin, 200 µg/ml streptomycin). The reconstitution mixture was cast in circular molds (450 µL/EHM) and further cultured in Iscove-Medium containing 4% B27 without insulin, 1% non-essential amino acids, 2 mmol/L glutamine, 200 µmol/L ascorbic acid, 100 U/ml penicillin, and 100 µg/ml streptomycin further supplemented with 100 ng/ml IGF1 (AF-100-11), 10 ng/ml FGF-2 (AF-100-18B), 5 ng/ml $VEGF_{165}$ (AF-100-20) and 5 ng/ml TGF-β1 (AF-100-21C; culture days 0-3 only). All growth factors were purchased from Peprotech.

Isometric force measurements. Force of contraction (FOC, i.e., systolic peak force minus diastolic tension) was measured under isometric conditions in organ baths at 37°C in gassed (5% CO_2/95% O_2) Tyrode's solution (containing in mmol/L: 120 NaCl, 1 $MgCl_2$, 0.2 $CaCl_2$, 5.4 KCl, 22.6 $NaHCO_3$, 4.2 NaH_2PO_4, 5.6 glucose, and 0.56 ascorbate) as previously described.[2] EHMs were analyzed at 1 Hz with 5 ms square pulses of 200 mA electrical current. EHMs were mechanically stretched at intervals of 125 µm until the maximum twitch force was observed (Lmax) at 2 mmol/L calcium. Subsequently, maximal inotropic capacity was investigated under increasing extracellular calcium concentrations (0.2-4 mmol/L).

Statistical Analyses. Data are displayed as means with standard error of the mean (SEM) unless stated otherwise. *n* indicates the number of sample/biological replicates analyzed. Statistical analyses were used for each data set as indicated in the Figure legends using Graph Pad Prism. A *P*-value of < 0.05 was considered as an indication for significant differences between investigated groups.

Synthesis Schemes

Scheme 1: Chalcones 1,2, and Synthesis of 5o

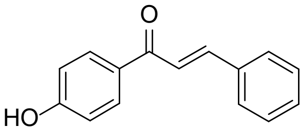

1

4'-Hydroxychalcone (4'HC)
(commercially available)

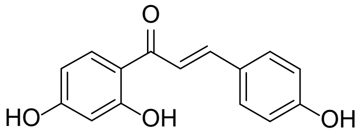

2

Isoquiritigenin (Iso)
(commercially available)

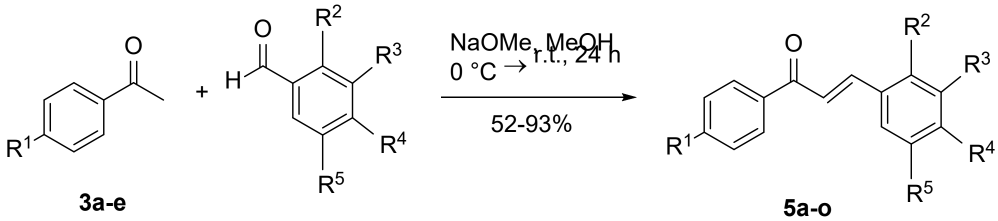

3a-e

4a-i

5a-o

**5**	a	b	c	d	e	f	g	h	i	j	k	l	m	n	o
R^1	OMe	OMe	OMe	Me	OH	OH	OMe	NO_2	F	NO_2	NO_2	NO_2	OMe	F	F
R^2	H	H	H	H	H	H	H	H	H	H	H	H	Br	H	H
R^3	H	H	H	H	H	H	H	H	H	H	H	H	H	OMe	OH
R^4	F	OMe	NAc H	F	OMe	NAc H	CO_2 Me	OMe	CO_2 Me	F	OH	Me	H	H	H
R^5	H	H	H	H	H	H	H	H	H	H	H	H	F	H	H

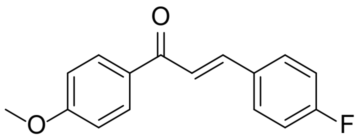

(*E*)-3-(4-Fluorophenyl)-1-(4-methoxyphenyl)prop-2-en-1-one (5a):

^{1}H NMR (300 MHz, $CDCl_3$): δ 3.87 (s, 3H), 6.93 – 7.01 (m, 2H), 7.00 – 7.12 (m, 2H), 7.46 (d, J = 15.6 Hz, 1H), 7.56 – 7.66 (m, 2H), 7.75 (d, J = 15.6 Hz, 1H), 7.98 – 8.08 (m, 2H).

^{13}C NMR (126 MHz, $CDCl_3$): δ 55.4 (CH_3), 113.8 (CH), 115.8 (CH), 116.0 (CH), 121.5 (CH), 130.11 (CH), 130.18 (CH), 130.71 (CH), 130.9 (CH), 131.26 (CH), 131.28 (C), 142.5 (CH), 162.8 (C), 164.8 (C), 163.4 (C), 188.3 (C=O).

**$C_{16}H_{13}FO_2$** (256.2716)	$[M+Na]^+$	calc.: 279.0791
	ESI-HRMS	found: 279.0792

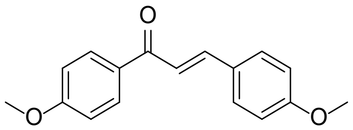

(*E*)-1,3-Bis(4-methoxyphenyl)prop-2-en-1-one (5b):

^{1}H NMR (500 MHz, $CDCl_3$): δ 3.85 (s, 3H), 3.88 (s, 3H), 6.91 – 6.95 (m, 2H), 6.96 – 7.00 (m, 2H), 7.43 (d, J = 15.6 Hz, 1H), 7.57 – 7.63 (m, 2H), 7.78 (d, J = 15.6 Hz, 1H), 8.00 – 8.06 (m, 2H).

^{13}C NMR (126 MHz, $CDCl_3$): δ 55.4 (OCH_3), 113.7 (CH), 114.3 (CH), 119.5 (C), 127.7 (C), 129.9 (CH), 130.5 (CH), 131.2 (C), 143.6 (C), 161.34 (C), 163.1 (C), 188.5 (C=O).

**$C_{17}H_{16}O_3$** (268.3071)	$[M+Na]^+$	calc.: 291.0993
	ESI-HRMS	found: 291.0992

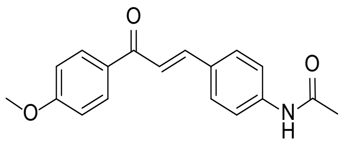

(*E*)-*N*-(4-(3-(4-Methoxyphenyl)-3-oxoprop-1-en-1-yl)phenyl)acetamide (5c):

^{1}H NMR (500 MHz, $CDCl_3$): δ 2.20 (s, 3H), 3.89 (s, 3H), 6.95 – 7.01 (m, 2H), 7.48 (d, *J* = 15.6 Hz, 1H), 7.55 (s, 1H), 7.54 – 7.61 (m, 4H), 7.75 (d, *J* = 15.6 Hz, 1H), 8.00 – 8.06 (m, 2H).

^{13}C NMR (126 MHz, $CDCl_3$): δ 24.7 (CH_3), 55.5 (OCH_3), 113.7 (CH), 119.6 (CH), 120.7 (CH), 129.2 (CH), 130.6 (C), 131.0 (C), 139.7 (C), 143.2 (CH), 163.2 (C), 168.2 (CO), 188.5 (CO).

**$C_{18}H_{17}NO_3$** (295.3325)	$[M+Na]^+$	calc.: 318.1103
	ESI-HRMS	found: 318.1101

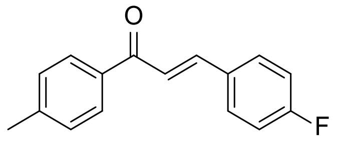

(*E*)-3-(4-Fluorophenyl)-1-(*p*-tolyl)prop-2-en-1-one (5d):

^{1}H NMR (500 MHz, $CDCl_3$): δ 2.44 (s, 3H), 7.06 – 7.15 (m, 2H), 7.31 (dt, *J* = 8.0, 0.8 Hz, 2H), 7.46 (d, *J* = 15.7 Hz, 1H), 7.59 – 7.67 (m, 2H), 7.77 (d, *J* = 15.7 Hz, 1H), 7.89 – 7.98 (m, 2H).

^{13}C NMR (126 MHz, $CDCl_3$): δ 21.7 (CH_3), 115.9 (CH), 116.0 (CH), 121.7 (CH), 128.5 (CH), 129.2 (CH), 130.1 (CH), 131.1 (C), 135.4 (CH), 142.9 (CH), 143.5 (C), 162.8 (C), 164.8 (C), 189.5 (CO).

**$C_{16}H_{13}FO$** (240.2722)	$[M+Na]^+$	calc.: 263.0838
	ESI-HRMS	found: 263.0843

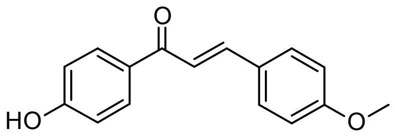

(*E*)-3-(4-Methoxyphenyl)-1-(4-hydroxyphenyl)prop-2-en-1-one (5e)[3]:

$C_{16}H_{14}O_3$ (254.2806)

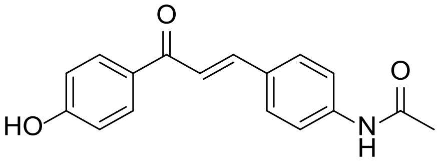

(*E*)-*N*-(4-(3-(4-Hzdroxyphenyl)-3-oxoprop-1-en-1-yl)phenyl)acetamide (5f):

^{1}H NMR (300 MHz, d_6-DMSO): δ 2.08 (s, 3H), 6.82 – 6.95 (m, 2H), 7.60 – 7.70 (m, 4H), 7.74 (d, *J* = 8.8 Hz, 2H), 7.92 – 8.08 (m, 2H), 9.95 (s, 1H).

^{13}C NMR (126 MHz, d_6-DMSO): δ 24.1 (CH_3), 115.3 (CH), 118.8 (CH), 120.2 (CH), 129.2 (CH), 129.3 (C), 129.4 (C), 130.9 (CH), 141.1 (C), 142.3 (CH), 161.8 (C), 168.5 (CO), 186.9 (CO).

**$C_{17}H_{15}NO_3$** (281.3059)	$[M+H]^+$	calc.: 282.1118
	ESI-HRMS	found: 282.1125

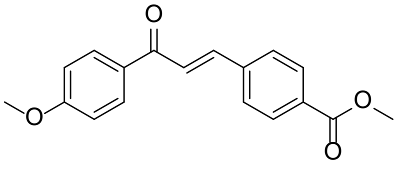

Methyl (*E*)-4-(3-(4-methoxyphenyl)-3-oxoprop-1-en-1-yl)benzoate (5g):

^{1}H NMR (400 MHz, $CDCl_3$): δ 3.90 (s, 3H), 3.94 (s, 3H), 6.96 – 7.04 (m, 2H), 7.62 (d, *J* = 15.7 Hz, 1H), 7.66 – 7.73 (m, 2H), 7.80 (d, *J* = 15.7 Hz, 1H), 8.02 – 8.12 (m, 4H).

^{13}C NMR (101 MHz, $CDCl_3$): δ 52.2 (OCH_3), 55.5 (OCH_3), 113.9 (CH), 123.9 (CH), 128.1 (CH), 130.1 (CH), 130.7 (C), 130.8 (CH), 131.3 (C), 139.3 (C), 142.3 (CH), 163.6 (C), 166.4 (OCO), 188.2 (CO).

**$C_{18}H_{16}O_4$** (296.3172)	$[M+Na]^+$	calc.: 319.0953
	ESI-HRMS	found: 319.091

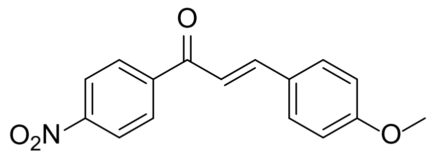

(*E*)-3-(4-Methoxyphenyl)-1-(4-nitrophenyl)prop-2-en-1-one (5h):

^{1}H NMR (500 MHz, $CDCl_3$): δ 3.88 (s, 3H), 6.93 – 6.99 (m, 2H), 7.36 (d, J = 15.6 Hz, 1H), 7.63 (d, J = 15.6 Hz, 1H), 7.79 – 7.86 (m, 1H), 8.10 – 8.16 (m, 2H), 8.32 – 8.38 (m, 2H).

^{13}C NMR (126 MHz, $CDCl_3$): δ 55.5 (OCH_3), 114.5 (CH), 118.9 (CH), 123.7 (CH), 123.9 (CH), 126.9 (CH), 129.2 (CH), 129.3 (C), 130.5 (CH), 143.3 (C), 146.6 (CH), 149.8 (C), 162.1 (C), 188.8 (CO).

**$C_{16}H_{13}NO_4$** (283.2787)	$[M+Na]^+$	calc.: 306.0728
	ESI-HRMS	found: 306.0737

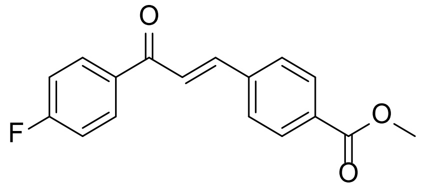

Methyl (*E*)-4-(3-(4-fluorophenyl)-3-oxoprop-1-en-1-yl)benzoate (5i):

^{1}H NMR (300 MHz, $CDCl_3$): δ 3.91 (s, 3H), 7.08 – 7.22 (m, 2H), 7.55 (d, J = 15.7 Hz, 1H), 7.66 (d, J = 8.3 Hz, 2H), 7.78 (d, J = 15.7 Hz, 1H), 8.05 (ddd, J = 8.8, 3.9, 2.1 Hz, 4H).

^{13}C NMR (126 MHz, $CDCl_3$): δ 52.2 (OCH_3), 115.6 (CH), 115.8 (CH), 123.5 (s), 126.5 (C), 128.1 (CH), 129.8 (CH), 130.0 (CH), 131.0 (CH), 131.5 (s), 134.1 (C), 138.8 (CH), 143.2 (CH), 164.6 (C), 166.2 (C), 166.6 (OC=O), 188.2 (C=O).

**$C_{17}H_{13}FO_3$** (284.2817)	$[M+Na]^+$	calc.: 307.0739
	ESI-HRMS	found: 307.0741

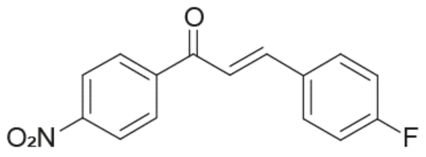

(*E*)-3-(4-Fluorphenyl)-1-(4-nitrophenyl)prop-2-en-1-one (5j)[4]:

$C_{15}H_{10}FNO_3$ (271.2432)

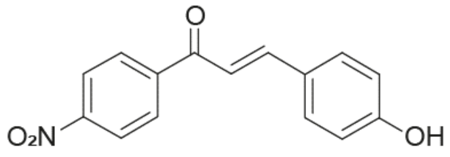

(*E*)-3-(4-Hydroxyphenyl)-1-(4-nitrophenyl)prop-2-en-1-one (5k)[5]:

$C_{15}H_{11}NO_4$ (269.2521)

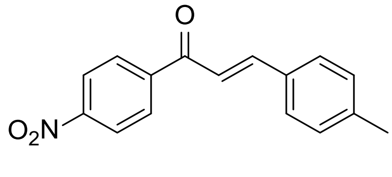

(*E*)-1-(4-Nitrophenyl)-3-(p-tolyl)prop-2-en-1-one (5l):

^{1}H NMR (500 MHz, $CDCl_3$): δ 2.41 (s, 3H), 7.25 (dt, *J* = 8.4, 0.8 Hz, 2H), 7.44 (d, *J* = 15.7 Hz, 1H), 7.53 – 7.59 (m, 2H), 7.83 (d, *J* = 15.7 Hz, 1H), 8.11 – 8.17 (m, 2H), 8.32 – 8.39 (m, 2H).

^{13}C NMR (126 MHz, $CDCl_3$): δ 21.6 (CH_3), 120.2 (CH), 123.7 (CH), 128.6 (CH), 129.2 (CH), 129.7 (CH), 131.5 (C), 141.8 (C), 143.1 (C), 146.8 (CH), 149.8 (C), 188.9 (CO).

**$C_{16}H_{13}NO_3$** (267.2793)	$[M+H]^+$	calc.: 268.0965
	ESI-HRMS	found: 268.0968

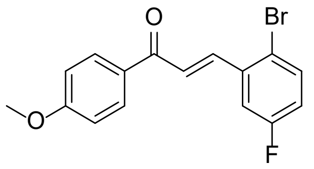

(*E*)-3-(2-Bromo-5-fluorophenyl)-1-(4-methoxyphenyl)prop-2-en-1-one (5m):

^{1}H NMR (500 MHz, $CDCl_3$): δ 3.89 (s, 3H), 6.94 – 7.02 (m, 3H), 7.38 – 7.45 (m, 2H), 7.58 (dd, *J* = 8.8, 5.3 Hz, 1H), 7.98 – 8.07 (m, 3H).

^{13}C NMR (126 MHz, $CDCl_3$): δ 55.5 (OCH_3), 113.8 (CH), 114.2 (CH), 114.4 (CH), 118.3 (CH), 119.8 (C), 125.7 (CH), 130.4 (CH), 130.8 (CH), 134.6 (CH), 136.8 (C), 140.9 (CH), 160.7 (C), 162.7 (C), 163.5 (C), 187.8 (CO).

**$C_{16}H_{12}BrFO_2$** (335.1677)	$[M+H]^+$	calc.: 335.0081
	ESI-HRMS	found: 335.0077

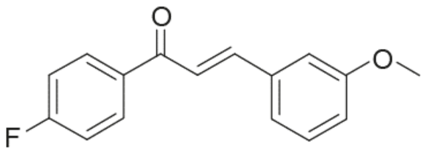

(*E*)-3-(3-Methoxyphenyl)-1-(4-fluorphenyl)prop-2-en-1-one (5n)[6]:

$C_{16}H_{13}FO_2$ (256.2716)

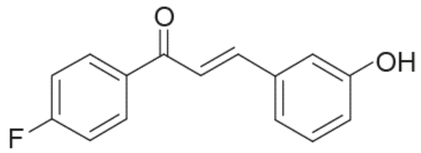

(*E*)-3-(3-Hydroxyphenyl)-1-(4-fluorphenyl)prop-2-en-1-one (5o)[5a, 7]:

$C_{15}H_{11}FO_2$ (242.2450)

Scheme 2: Synthesis of 6a-c

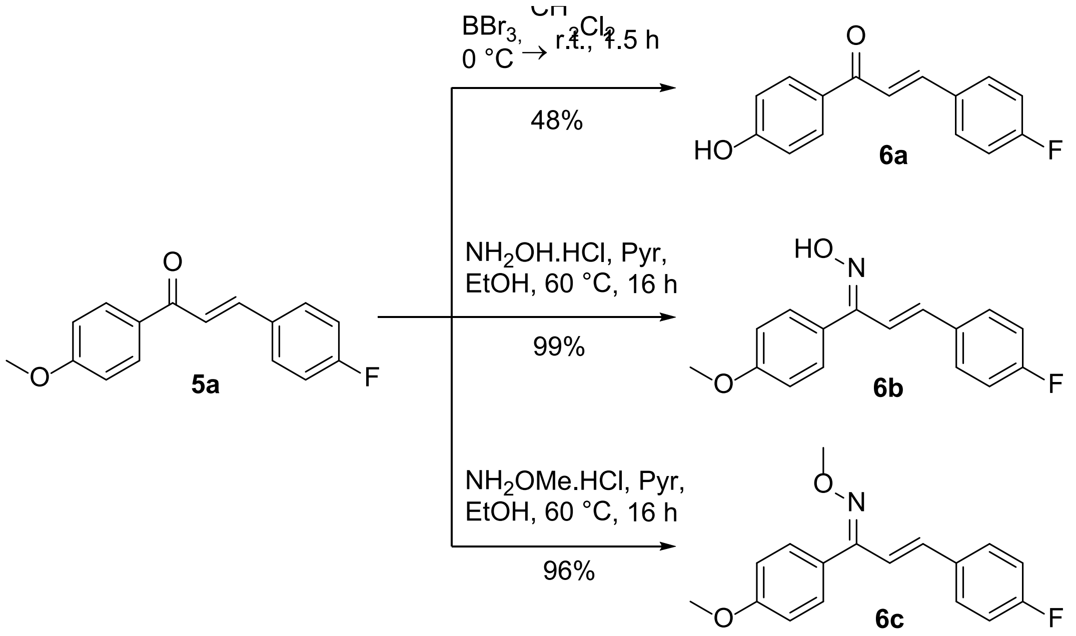

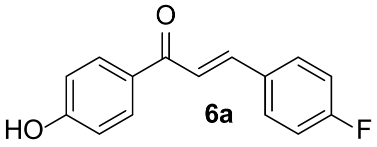

(2*E*)-3-(4-Fluorophenyl)-1-(4-hydroxyphenyl)prop-2-en-1-one (6a)[10]: BBr_3 (2.34 mL, 2.34 mmol) in CH_2Cl_2 (3 mL) was added dropwise to a solution of **5a**[11] (200 mg, 0.78 mmol) in CH_2Cl_2 (3 mL) at 0ºC. The reaction mixture was stirred at room temperature for 1.5 h and the reaction quenched with MeOH at 0ºC, then the mixture was diluted with water and extracted with CH_2Cl_2 (3 x). The organic phase was dried (Na_2SO_4) and concentrated. The residue was purified by column chromatography on silica gel using 5 – 20% ethyl acetate in petroleum ether to obtain compound **6a** (91 mg, Yield: 48%) as a light orange solid.

^{1}H NMR (300 MHz, DMSO-*d*$_6$) δ 10.36 (s, 1H), 8.06 (d, *J* = 8.7 Hz, 2H), 7.97 – 7.89 (m, 2H), 7.85 (d, *J* = 15.6 Hz, 1H), 7.67 (d, *J* = 15.6 Hz, 1H), 7.27 (t, *J* = 8.8 Hz, 2H), 6.90 (d, *J* = 8.7 Hz, 2H).

^{13}C NMR (126 MHz, DMSO-*d*$_6$) δ 186.78, 163.92, 161.95, 141.15, 131.35, 130.90, 130.70, 128.91, 121.92, 115.61, 115.16.

**$C_{15}H_{11}FO_2$** (242.2450)	$[M+Na]^+$	calc.:	265.0635
	ESI-HRMS	found:	265.0635

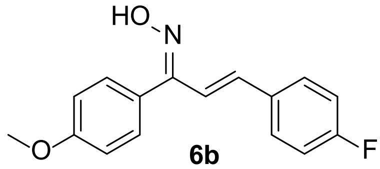

6b

***N*-[(1*Z*,2*E*)-3-(4-Fluorophenyl)-1-(4-methoxyphenyl)prop-2-en-1-ylidene]hydroxylamine (6b)[12]:** A mixture of compound **5a** (300 mg, 1.17 mmol), $NH_2OH{\cdot}HCl$ (244 mg, 3.51 mmol) and pyridine (1 mL) in EtOH (10 mL) was heated at 60°C for 16 h. Then the mixture was concentrated in vacuo, the residue acidified with 1N aq HCl and extracted with ethyl acetate (3 x). The organic phase was dried (Na_2SO_4) and evaporated in vacuo. The obtained crude material was purified by column chromatography on silica gel using 5 – 15% ethyl acetate in petroleum ether to get compound **6b** (314 mg, Yield: 99%) as a white solid.

^{1}H NMR (300 MHz, $CDCl_3$) δ 7.57 (d, *J* = 16.5 Hz, 1H), 7.50 – 7.40 (m, 4H), 7.07 – 6.97 (m, 2H), 6.97 – 6.91 (m, 2H), 6.77 (d, *J* = 16.5 Hz, 1H), 3.84 (s, 3H).

**$C_{16}H_{14}FNO_2$** (271.2863)	$[M+H]^+$	calc.: 272.1081
	ESI-HRMS	found: 272.1081

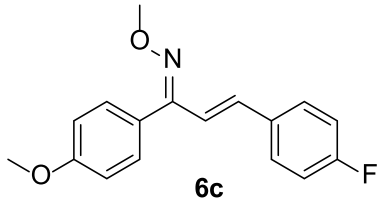

6c

(1*Z*,2*E*)-3-(4-Fluorophenyl)-*N*-methoxy-1-(4-methoxyphenyl)prop-2-en-1-imine (6c): A mixture of compound **5a** (300 mg, 1.17 mmol), $MeONH_2{\cdot}HCl$ (293 mg, 3.51 mmol) and pyridine (1 mL) in EtOH (10 mL) was heated at 60°C for 16 h. The mixture was concentrated in vacuo, the obtained residue acidified with 1N aq HCl and extracted with ethyl acetate (3 x). The organic phase was dried (Na_2SO_4) and evaporated in vacuo. The obtained crude material was purified by column chromatography on silica gel using 5 – 15% ethyl acetate in pet ether to get compound **6c** (321 mg, Yield: 96%) as a white solid.

^{1}H NMR (300 MHz, $CDCl_3$) δ 7.53 – 7.40 (m, 4H), 7.30 – 7.22 (m, 1H), 7.06 – 6.89 (m, 4H), 6.73 (d, *J* = 16.6 Hz, 1H), 4.04 (s, 3H), 3.83 (s, 3H).

**$C_{17}H_{16}FNO_2$** (285.3128)	$[M+H]^+$	calc.: 286.1239
	ESI-HRMS	found: 286.1238

Scheme 3: Synthesis of 6d and 6e

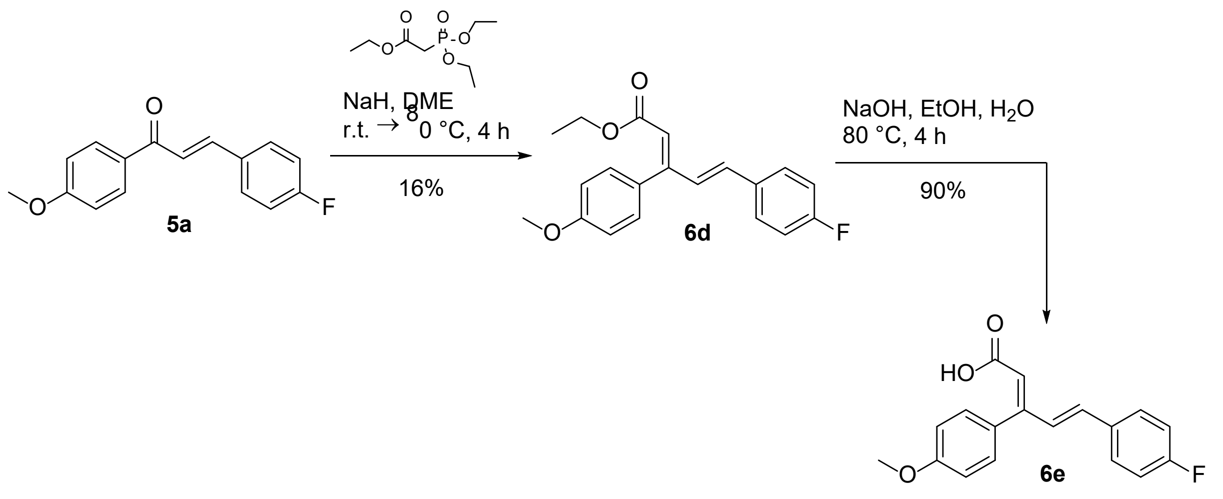

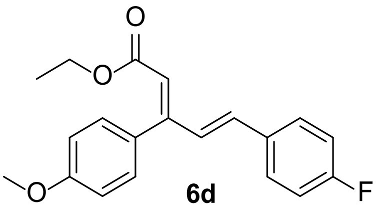

Ethyl (2*Z*,4*E*)-5-(4-fluorophenyl)-3-(4-methoxyphenyl)penta-2,4-dienoate (6d): A solution of triethylphosphonoacetate (874 mg, 3.9 mmol) in dimethoxyethane (1 mL) and a solution of compound **5a** (1 g, 3.9 mmol) in dimethoxyethane (7 mL) were successively added to a suspension of 60% NaH (156 mg, 3.9 mmol) in dimethoxyethane (7 mL) at room temperature. The reaction mixture was heated at 80°C for 4 h, then cooled to room temperature, diluted with water (100 mL), and extracted with CH_2Cl_2 (3 x). The organic phase was dried (Na_2SO_4) and evaporated in vacuo. The obtained crude material was purified by column chromatography on silica gel using 5 – 10% ethyl acetate in petroleum ether to afford compound **6d** (206 mg, Yield: 16%) as a colourless thick liquid.

^{1}H NMR (300 MHz, $CDCl_3$) δ 8.40 (dt, J = 16.2, 0.8 Hz, 1H), 7.50 – 7.38 (m, 2H), 7.30 (d, J = 8.7 Hz, 2H), 6.99 (t, J = 8.7 Hz, 2H), 6.91 (d, J = 8.7 Hz, 2H), 6.59 (d, J = 16.2 Hz, 1H), 5.78 (d, J = 0.9 Hz, 1H), 4.22 (q, J = 7.1 Hz, 2H), 3.84 (s, 3H), 1.32 (t, J = 7.1 Hz, 3H).

^{13}C NMR (126 MHz, $CDCl_3$) δ 166.35, 163.84, 161.86, 159.92, 155.27, 137.90, 132.87, 132.12, 130.19, 128.96, 126.00, 117.03, 115.59, 113.63, 59.96, 55.33, 14.45.

**$C_{20}H_{19}FO_3$** (326.3615)	$[M+Na]^+$	calc.:	349.1205
	ESI-HRMS	found:	349.1210

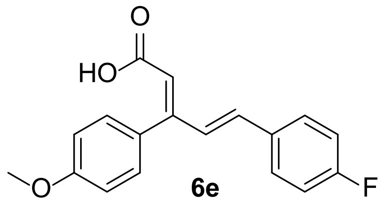

6e

(2*Z*,4*E*)-5-(4-Fluorophenyl)-3-(4-methoxyphenyl)penta-2,4-dienoic acid (6e): A mixture of compound **6d** (120 mg, 0.37 mmol) and NaOH (147 mg, 3.67 mmol) in EtOH (6 mL) and water (1.5 mL) was heated at 80ºC for 4 h. The reaction mixture was cooled to room temperature, acidified with 2N HCl, extracted with ethyl acetate (3 x). The organic phase was dried (Na_2SO_4) and concentrated under vacuum. The resulting solid was washed with pet ether to get the product **6e** (97 mg, Yield: 90%) as a white solid.

^{1}H NMR (300 MHz, DMSO-d_6) δ 12.27 (s, 1H), 8.29 (d, *J* = 16.3 Hz, 1H), 7.53 (d, *J* = 8.7, 2H), 7.33 (d, *J* = 8.6 Hz, 2H), 7.21 (t, *J* = 8.9 Hz, 2H), 7.01 (d, *J* = 8.7 Hz, 2H), 6.59 (d, *J* = 16.3 Hz, 1H), 5.72 (s, 1H), 3.81 (s, 3H).

^{13}C NMR (126 MHz, DMSO-d_6) δ 166.68, 163.01, 161.05, 159.48, 153.50, 136.40, 132.69, 131.28, 129.83, 128.85, 125.83, 117.88, 115.59, 113.74, 55.07.

**$C_{18}H_{15}FO_3$** (298.3083)	$[M+Na]^+$	calc.: 321.0893
	ESI-HRMS	found: 321.0897

Scheme 4: Synthesis of 6f and 6g

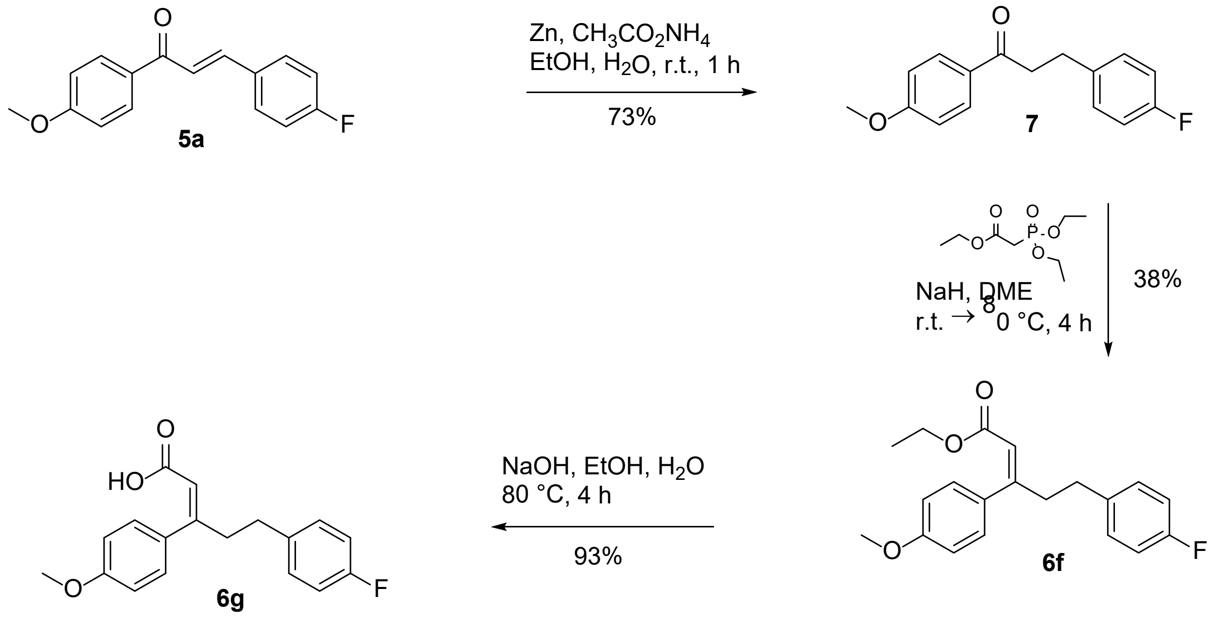

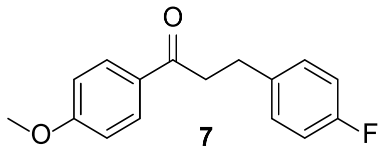

3-(4-Fluorophenyl)-1-(4-methoxyphenyl)propan-1-one (7)[13]: To a solution of **5a** (200 mg, 0.78 mmol) in EtOH (50 mL) was added a solution of ammonium acetate (6 g, 78 mmol) in water (8 mL). Then zinc powder (382 mg, 5.85 mmol, 7.5 eq) was put in at room temperature in 5 portions at intervals of 15 min. After completion of the reaction within 70 min (monitored by TLC), the mixture was filtered, the filter cake washed with EtOH (2 x 15 mL), and the combined filtrates concentrated in vacuo. The residue was diluted with water, extracted with ethyl acetate (3 x) and the organic phase was dried (Na_2SO_4) and concentrated in vacuo. Crude product was purified by column chromatography on silica gel using 5% ethyl acetate in pet ether to obtain compound **7** (133 mg, Yield: 73%) as a colourless thick liquid.

^{1}H NMR (300 MHz, $CDCl_3$) δ 7.99 – 7.79 (m, 2H), 7.27 – 7.06 (m, 2H), 7.03 – 6.79 (m, 4H), 3.84 (s, 3H), 3.20 (ddd, J = 8.0, 6.9, 0.9 Hz, 2H), 3.01 (t, J = 7.5 Hz, 3H).

^{13}C NMR (126 MHz, $CDCl_3$) δ 197.33, 163.33, 162.17, 160.23, 136.93, 130.15, 129.81, 129.69, 115.10, 113.65, 55.45, 40.07, 29.49.

**$C_{16}H_{15}FO_2$** (258.2875)	$[M+Na]^+$	clac.: 281.0949
	ESI-HRMS	found: 281.0948

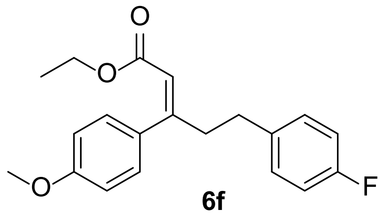

6f

Ethyl (2*Z*)-5-(4-fluorophenyl)-3-(4-methoxyphenyl)pent-2-enoate (6f): Triethylphosphonoacetate (1.15 mL, 5.8 mmol) was added to a suspension of 60% NaH (232 mg, 5.8 mmol) in dimethoxyethane (20 mL) at room temperature and the mixture stirred for 1 h. A solution of compound **7** (500 mg, 1.93 mmol) in dimethoxyethane (5 mL) was slowly added at room temperature and the reaction mixture heated at 80°C for 4 h. After cooling to room temperature, the mixture was diluted with water (100 mL) and extracted with CH_2Cl_2 (3 x). The organic phase was dried (Na_2SO_4) and evaporated in vacuo. The obtained crude material was purified by column chromatography on silica gel using 5 – 10% ethyl acetate in petroleum ether to afford compound **6f** (241 mg, Yield: 38%) as a colourless thick liquid.

^{1}H NMR (300 MHz, $CDCl_3$) δ 7.40 (d, J = 9.0 Hz, 2H), 7.20 – 7.11 (m, 2H), 6.97 – 6.85 (m, 4H), 6.02 (s, 1H), 4.17 (q, J = 7.1 Hz, 2H), 3.83 (s, 3H), 3.40 – 3.26 (m, 2H), 2.75 – 2.62 (m, 2H), 1.29 (t, J = 7.1 Hz, 3H).

^{13}C NMR (126 MHz, $CDCl_3$) δ 166.33, 162.17, 160.35, 158.43, 137.14, 132.91, 129.76, 127.94, 116.09, 114.86, 113.97, 59.79, 55.37, 34.55, 32.95, 14.44.

**$C_{20}H_{21}FO_3$** (328.3773)	$[M+Na]^+$	calc.: 351.1369
	ESI-HRMS	found: 351.1367

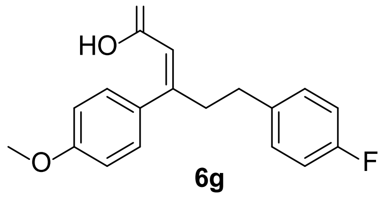

6g

(2*Z*)-5-(4-Fluorophenyl)-3-(4-methoxyphenyl)pent-2-enoic acid (6g): A mixture of **6f** (61 mg, 0.185 mmol) and NaOH (74 mg, 1.85 mmol) in EtOH (4 mL) and water (1 mL) was heated at 80°C for 4 h. The reaction mixture was cooled to room temperature, acidified with 2N HCl, and extracted with ethyl acetate (3 x). The organic phase was dried (Na_2SO_4) and concentrated

in vacuo. The resulting solid was washed with petroleum ether to get the desired compound **6g** (51 mg, Yield: 93%) as a white solid.

^{1}H NMR (300 MHz, DMSO-d_6) δ 12.06 (s, 1H), 7.52 (d, J = 8.8 Hz, 2H), 7.22 (dd, J = 8.6, 5.7 Hz, 2H), 7.07 (t, J = 8.9 Hz, 2H), 6.98 (d, J = 8.8 Hz, 2H), 6.00 (s, 1H), 3.80 (s, 3H), 3.37 – 3.26 (m, 2H), 2.69 – 2.57 (m, 2H).

^{13}C NMR (126 MHz, DMSO-d_6) δ 166.91, 161.32, 159.80, 159.41, 156.88, 137.18, 131.93, 129.64, 127.75, 116.16, 114.60, 113.92, 55.10, 33.90, 31.60.

**$C_{18}H_{17}FO_3$** (300.3242)	$[M+Na]^+$	calc.:	323.1050
	ESI-HRMS	found:	323.1054

Scheme 5: Synthesis of 6h

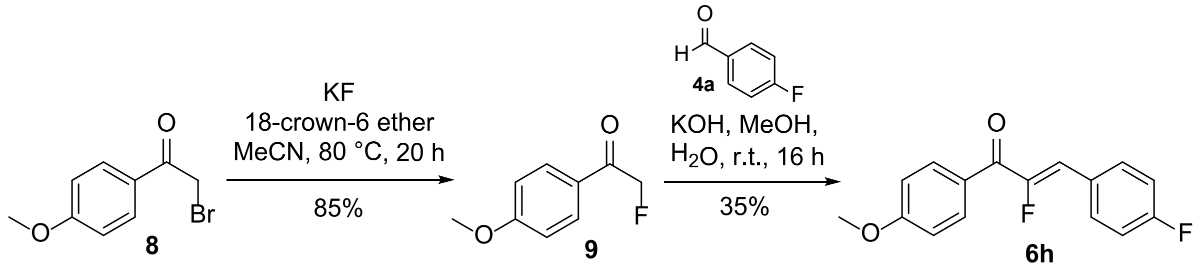

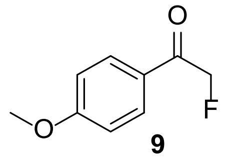

9

2-Fluoro-1-(4-methoxyphenyl)ethan-1-one (9)[14]: A mixture of 2-bromo-4-methoxyacetophenone (500 mg, 2.18 mmol; labelled as compound **8**), KF (634 mg, 10.9 mmol) and 18-crown-6 ether (115 mg, 0.44 mmol) in CH_3CN (10 mL) was heated at 80ºC for 20 h under Argon atmosphere. The reaction mixture was concentrated and the residue was diluted with water, extracted with ethyl acetate (3 x). Combined organic layer was washed with brine, dried (Na_2SO_4), and concentrated under vacuum. Crude product was purified on silica gel column chromatography using 0 – 5% ethyl acetate in pet ether to get the desired compound **9** (310 mg, Yield: 85%) as a white solid.

^{1}H NMR (300 MHz, $CDCl_3$) δ 7.93 – 7.78 (m, 2H), 7.03 – 6.82 (m, 2H), 5.56 – 5.37 (m, 2H), 3.86 (s, 3H).

$C_9H_9FO_2$ (168.1650)

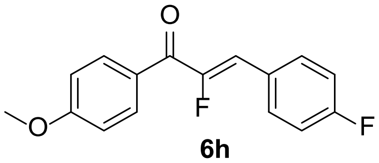

6h

(2*Z*)-2-Fluoro-3-(4-fluorophenyl)-1-(4-methoxyphenyl)prop-2-en-1-one (6h): 20% aq KOH (0.1 mL) was added to a solution of compound **9** (100 mg, 0.59 mmol) and 4-fluorobenzaldehyde (73.5 mg, 0.59 mmol) in MeOH (2 mL) at room temperature and the mixture was stirred at room temperature for 16 h. Ice cold water was added and the reaction mixture acidified with 2N aq HCl. The resulting precipitate was filtered off, washed with water

(4 x 7 mL) and allowed to dry at the air. The product was purified by column chromatography on silica gel using 5% ethyl acetate in petroleum ether to afford compound **6h** (56 mg, Yield: 35%) as a white solid.

^{1}H NMR (300 MHz, $CDCl_3$) δ 7.96 – 7.90 (m, 2H), 7.72 – 7.63 (m, 2H), 7.09 (t, J = 8.7 Hz, 2H), 6.96 (d, J = 8.9 Hz, 2H), 6.82 (d, J = 36.4 Hz, 1H), 3.87 (s, 3H).

^{13}C NMR (126 MHz, $CDCl_3$) δ 185.61, 164.08, 163.53, 162.08, 155.69, 153.51, 132.36, 131.84, 128.52, 127.73, 117.37, 115.93, 113.73, 55.52.

**$C_{16}H_{12}F_2O_2$** (274.2621)	$[M+Na]^+$	calc.:	297.0700
	ESI-HRMS	found:	297.0698

Scheme 6: Synthesis of 6i

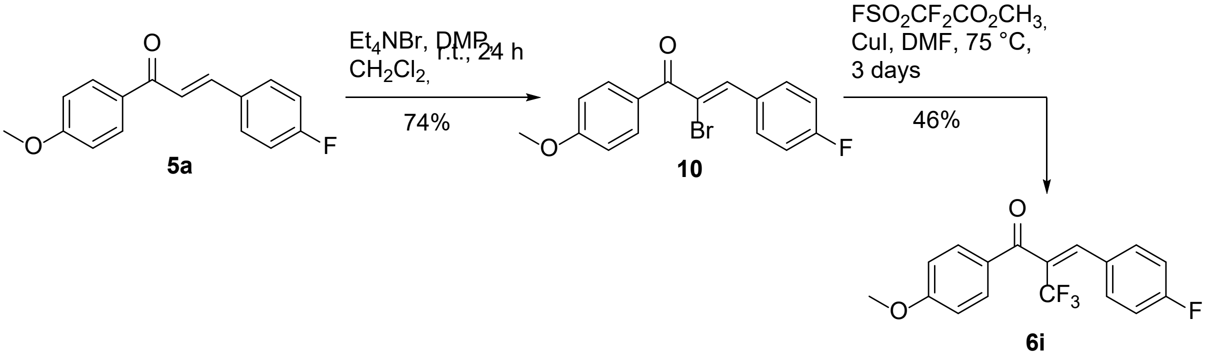

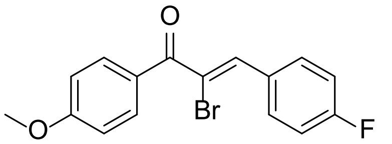

10

(2*Z*)-2-Bromo-3-(4-fluorophenyl)-1-(4-methoxyphenyl)prop-2-en-1-one (10): Et_4NBr (984 mg, 4.68 mmol) was added to a stirred suspension of Dess–Martin periodinane (DMP) (1.98 g, 4.68 mmol) in CH_2Cl_2 (8 mL) at room temperature and stirring was continued for 10 min with formation of a pale yellow solution. Compound **5a** (1 g, 3.9 mmol) was added to the mixture in portions at room temperature and stirring was continued at room temperature for 24 h. Then the mixture was diluted with CH_2Cl_2 (50 mL), washed with a sat solution of $NaHSO_3$ and a sat solution of $NaHCO_3$ and then with water. The organic phase was dried (Na_2SO_4) and concentrated in vacuo. Purification by column chromatography on silica gel using 5% ethyl acetate in petroleum ether yielded compound **10** (970 mg, Yield: 74%) as a pale-yellow thick liquid.

^{1}H NMR (300 MHz, $CDCl_3$) δ 7.96 – 7.89 (m, 2H), 7.25 (s, 1H), 7.18 – 7.09 (m, 2H), 6.91 – 6.82 (m, 4H), 3.83 (s, 3H).

^{13}C NMR (126 MHz, $CDCl_3$) δ 190.34, 164.49, 163.39, 161.40, 133.91, 132.37, 129.88, 126.09, 115.64, 114.21, 113.81, 55.56.

**$C_{16}H_{12}BrFO_2$** (335.1677)	$[M+Na]^+$	calc.:	356.9886
	ESI-HRMS	found:	356.9897

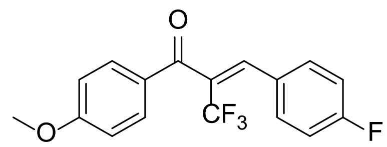

6i

(2*Z*)-3-(4-Fluorophenyl)-1-(4-methoxyphenyl)-2-(trifluoromethyl)prop-2-en-1-one (6i): A mixture of compound **10** (200 mg, 0.7 mmol), CuI (136 mg, 0.72 mmol) and methyl 2,2-difluoro-2-(fluorosulfonyl) acetate (0.55 mL, 4.35 mmol) in dry DMF (20 mL) was heated at 75ºC for 3 days under an argon atmosphere. Then, the reaction mixture was cooled to room temperature, diluted with ether (50 mL), filtered, the filter cake washed with ether and the combined filtrates mixed with water (150 mL) and extracted with ether. The organic phase was washed with brine, dried (Na_2SO_4) and concentrated in vacuo. Purification by column chromatography on silica gel using 10% ethyl acetate in petroleum ether yielded compound **6i** (89 mg, Yield: 46%) as colourless thick liquid.

^{1}H NMR (300 MHz, $CDCl_3$) δ 7.85 (d, *J* = 9.0 Hz, 2H), 7.36 (d, *J* = 1.8 Hz, 1H), 7.29 – 7.19 (m, 2H), 6.92 – 6.79 (m, 4H), 3.81 (s, 3H).

^{13}C NMR (126 MHz, Chloroform-*d*) δ 190.61, 164.54, 164.29, 162.29, 134.63 (q, *J* = 5.9 Hz), 132.03, 131.46 (d, *J* = 8.6 Hz), 128.33, 128.24 (d, *J* = 3.4 Hz), 123.29, 121.12, 115.85 (d, *J* = 21.9 Hz), 114.12, 55.53.

**$C_{17}H_{12}F_4O_2$** (324.2696)	$[M+Na]^+$	calc.: 347.0667
	ESI-HRMS	found: 347.0666

References

[1] S. Irion, H. Luche, P. Gadue, H. J. Fehling, M. Kennedy, G. Keller, *Nature biotechnology* **2007**, *25*, 1477-1482.

[2] M. Tiburcy, J. E. Hudson, P. Balfanz, S. Schlick, T. Meyer, M. L. Chang Liao, E. Levent, F. Raad, S. Zeidler, E. Wingender, J. Riegler, M. Wang, J. D. Gold, I. Kehat, E. Wettwer, U. Ravens, P. Dierickx, L. W. van Laake, M. J. Goumans, S. Khadjeh, K. Toischer, G. Hasenfuss, L. A. Couture, A. Unger, W. A. Linke, T. Araki, B. Neel, G. Keller, L. Gepstein, J. C. Wu, W. H. Zimmermann, *Circulation* **2017**, *135*, 1832-1847.

[3] aV. V. Truong, T. D. Nam, T. N. Hung, N. T. Nga, P. M. Quan, L. V. Chinh, S. H. Jung, *Bioorg Med Chem Lett* **2015**, *25*, 5182-5185; bS. Garg, N. Raghav, *Rsc Adv* **2015**, *5*, 72937-72949; cX. L. Yang, W. Wang, J. Tan, D. D. Song, M. Li, D. Liu, Y. K. Jing, L. X. Zhao, *Bioorganic & Medicinal Chemistry Letters* **2009**, *19*, 4385-4388; dF. Yesilyurt, A. Aydin, H. I. Gul, M. Akkurt, N. D. Ozcelik, *Acta Crystallogr E Crystallogr Commun* **2018**, *74*, 960-963.

[4] aR. A. Gupta, S. G. Kaskhedikar, *Med Chem Res* **2013**, *22*, 3863-3880; bY. L. Zhu, Y. J. Pan, *Chem Lett* **2004**, *33*, 668-669.

[5] aD. Moi, A. Nocentini, A. Deplano, G. Balboni, C. T. Supuran, V. Onnis, *Eur J Med Chem* **2019**, *182*, 111638; bA. S. Sherikar, R. P. Dhavale, M. S. Bhatia, *New J Chem* **2018**, *42*, 14365-14385.

[6] C. T. Hsieh, T. J. Hsieh, M. El-Shazly, D. W. Chuang, Y. H. Tsai, C. T. Yen, S. F. Wu, Y. C. Wu, F. R. Chang, *Bioorg Med Chem Lett* **2012**, *22*, 3912-3915.

[7] aC. P. Shah, P. S. Kharkar, *European Journal of Medicinal Chemistry* **2018**, *158*, 286-301; bX. Bai, W. Q. Shi, H. F. Chen, P. Zhang, Y. Li, S. F. Yin, *Chem Nat Compd+* **2012**, *48*, 60-65.

[8] K. J. Livak, T. D. Schmittgen, *Methods (San Diego, Calif.)* **2001**, *25*, 402-408.

[9] S. Tohyama, F. Hattori, M. Sano, T. Hishiki, Y. Nagahata, T. Matsuura, H. Hashimoto, T. Suzuki, H. Yamashita, Y. Satoh, T. Egashira, T. Seki, N. Muraoka, H. Yamakawa, Y. Ohgino, T. Tanaka, M. Yoichi, S. Yuasa, M. Murata, M. Suematsu, K. Fukuda, *Cell Stem Cell* **2013**, *12*, 127-137.

[10] B. I. Roman, T. De Ryck, A. Patronov, S. H. Slavov, B. W. A. Vanhoecke, A. R. Katritzky, M. E. Bracke, C. V. Stevens, *European Journal of Medicinal Chemistry* **2015**, *101*, 627-639.

[11] aS. T. Asundaria, K. C. Patel, *Pharm Chem J+* **2012**, *45*, 725-731; bM. D. Bowman, M. M. Jacobson, H. E. Blackwell, *Organic Letters* **2006**, *8*, 1645-1648.

[12] Y. Luo, R. Song, Y. Li, S. Zhang, Z. J. Liu, J. Fu, H. L. Zhu, *Bioorganic & Medicinal Chemistry Letters* **2012**, *22*, 3039-3043.

[13] aV. Kumar, A. Sharma, A. K. Sinha, *Helv Chim Acta* **2006**, *89*, 483-495; bZ. L. Xu, M. Y. Ba, H. Zhou, Y. L. Cao, C. J. Tang, Y. Yang, R. C. He, Y. Liang, X. M. Zhang, Z. Z. Li, L. H. Zhu, Y. Guo, C. B. Guo, *European Journal of Medicinal Chemistry* **2014**, *85*, 27-42.

[14] W. Borzecka, I. Lavandera, V. Gotor, *J Org Chem* **2013**, *78*, 7312-7317.
